# State capacity and health system financing: a cross-country analysis

**DOI:** 10.1136/bmjgh-2025-020101

**Published:** 2026-03-24

**Authors:** Sumit Mazumdar, Akseer Hussain, Marc Suhrcke, Kanksha Barman, Cameron Feil, Zaad Mahmood

**Affiliations:** 1 Centre for Health Economics, University of York, York, UK; 2 Luxembourg Institute of Socio-economic Research, Belval, Luxembourg; 3 Institute of Economic Growth, Delhi, Delhi, India; 4 Charité - Universitätsmedizin Berlin, Berlin, Germany; 5 Presidency University Kolkata, Kolkata, West Bengal, India

**Keywords:** Global Health, Health systems, Health economics

## Abstract

**Introduction:**

Achieving universal health coverage (UHC) requires not only financial resources but also strong and capable states that can mobilise, allocate and effectively manage those resources. Although fiscal capacity is widely acknowledged as a key determinant of health systems financing, state capacity is a broader, multidimensional construct that encompasses the administrative, legal and coercive functions of the state.

**Methods:**

This study investigates how multiple dimensions of state capacity—bureaucratic quality, corruption, rule of law, military involvement in politics, government effectiveness, property rights and state fragility—are associated with key measures of health financing. We analyse an unbalanced global panel of 141 countries, including 49 low- and middle-income countries, over the period 2000–2020. Using data from established cross-country institutional and health financing sources, we estimate fixed-effects and random-effects panel regression models to assess the relationship between state capacity and the two health financing metrics: government health expenditure per capita and out-of-pocket health spending as a share of current health expenditure, used here as a proxy for financial protection.

**Results:**

Our findings indicate that stronger state capacity is consistently associated with higher public health investment and reduced out-of-pocket spending by households. A 1 SD increase in bureaucratic quality is associated with a 2.6 percentage-point lower share of OOP health expenditure in current health spending. Similarly, 1 SD improvements in government effectiveness and property rights are associated with 1.6 and 2.8 percentage-point lower OOP shares, respectively. A 1 SD increase in rule of law, government effectiveness or property rights is associated with a 13%–31% higher level of government health expenditure (GHE) per capita, whereas a 1 SD increase in state fragility is associated with a 32% lower GHE per capita. The aggregate state capacity index is positively associated with GHE per capita, with a 1 SD increase corresponding to a 17.5% higher level of public health spending.

**Conclusion:**

The results underscore the critical role of state institutions in achieving sustainable and equitable health financing and highlight the importance of governance reforms in accelerating progress toward UHC across diverse national contexts.

**WHAT IS ALREADY KNOWN ON THIS TOPIC**

- Higher state capacity leads to better health outcomes, such as reduced mortality and disease prevalence.
- Different aspects of state capacity have been found to be individually associated with better health system performance and higher levels of public spending on health.

**WHAT THIS STUDY ADDS**

- Introduces multidimensional measures of state capacity and examines both their individual and combined associations with key indicators of health system financing.
- Consistent evidence of higher bureaucratic quality, control of corruption, civilian control of governments and stable property rights encouraging higher public spending on health and reducing out-of-pocket expenditure.

**HOW THIS STUDY MIGHT AFFECT RESEARCH, PRACTICE OR POLICY**

- Improving bureaucratic quality and reducing corruption improves state capacities that translate into greater financial protection in healthcare.
- Stronger institutional quality is associated with more effective public spending and stronger health systems financing.

## Introduction

 State capacity, broadly defined as a country’s ability to implement its policies and achieve its goals, plays a crucial role in long-term economic development and effective governance.^1 2^ Beyond these fundamental functions, state capacity also influences a range of social, political and economic outcomes. In contemporary literature, it is commonly understood as the state’s ability to implement policies and decisions to achieve specific objectives.^3^,^6^ Given its far-reaching implications, state capacity has received significant attention, highlighting its central role in shaping national progress and stability.

Empirical studies have shown positive associations between state capacity and economic growth,^2 7^ democratisation,^3^ regime stability^8^ and public goods provision.^9^ It also enhances welfare state generosity,^10^ protection of human rights^11^ and property rights,^12 13^ and resolves coordination failures.^14^ Additionally, state capacity contributes to human capital development, such as lowering child mortality^15 16^ and improving health and education outcomes.^17^ High state capacity is also associated with positive pandemic outcomes,^18 19^ better management of international conflicts and civil wars,^20 21^ and successful peace agreements.^22^

Despite a growing number of empirical studies, there is considerable disagreement on how to measure state capacity in cross-country research.^4^ Studies have used various proxies to operationalise this concept, including judgement-based indicators like the Varieties of Democracy’s Rigorous and Impartial Public Administration Index,^23^ Transparency International’s Corruption Perceptions Index^11^ and the World Bank’s Government Effectiveness metric.^18^ Other studies rely on observational proxies such as life expectancy, operating in two ways: first, as a reliable indicator of inequality and development, making it a strong proxy for gross domestic product (GDP) growth; and second, through the lens of enduring internal rivalry, where higher life expectancy increases the opportunity costs for rebels when deciding whether to initiate or recommit to fighting.^24 25^ Similarly, GDP per capita^2^ and fiscal capacity indicators such as tax revenue^8^ are frequently employed as structural proxies for underlying state capacity.

Consequently, this diversity of approaches reflects the fact that state capacity is not a single construct but a combination of core functions. The literature broadly conceptualises state capacity along three foundational dimensions: the ability to maintain territorial control and public order, the ability to extract resources to finance state activities and the administrative ability to deliver public goods and services.^3 26^ These are often referred to as military, fiscal and administrative capacities, respectively.^16^

The diverse range of state capacity measurements presents three key challenges: first, selecting appropriate state capacity indicators for a specific outcome of interest; second, drawing accurate inferences from these indicators to inform relevant policy decisions^27^; and third, strength in one area of state capacity (eg, coercive power) does not guarantee better state capacity in other areas (eg, bureaucratic or administrative functions). Many low- and middle-income countries (LMICs), see the classification in online supplemental table A18, demonstrate strong policing or military power but often lack the administrative capacity needed to effectively deliver public goods.^16 23 28^ While Savoia and Sen^1^ suggest that various measures of state capacity are highly correlated, Vaccaro^27^ argues that even strongly correlated indicators can yield different inferences, underscoring the complexities involved in measuring state capacity.

The relationship between state capacity and health system financing is critical, as governance quality directly influences public health spending and access to healthcare services. Strong fiscal capacity has been shown to improve health outcomes by facilitating more efficient resource allocation, especially in underserved or rural areas.^29 30^

However, to the best of our knowledge, the influence of different state capacity measures in health system financing remains under-explored. Previous research has consistently shown that higher tax revenues create the necessary fiscal space for increased public health expenditures, allowing governments to prioritise and finance healthcare services.^31^ Conversely, weak economic growth has been identified as a key factor behind low tax revenues and inadequate government spending on healthcare in LMICs.^32^

In this study, we examine how different indicators of state capacity—including bureaucratic quality, control of corruption, rule of law, military involvement in politics, government effectiveness, property rights and state fragility—influence the financing of the health system. These indicators of state capacity play distinct roles. Bureaucratic quality ensures stable policy implementation and reduces inefficiencies.^16^ Control of corruption minimises misallocation of funds, improving healthcare access.^33^ Rule of law promotes legal frameworks that protect healthcare investments.^34^ Military involvement in politics can undermine democratic accountability and healthcare priorities.^35^ Government effectiveness strengthens public service delivery.^36^ Stability of property rights encourages long-term investment in health infrastructure.^37^ State fragility has been associated with lower public health spending and instability.^38^ By analysing these interconnected indicators, we gain insight into how state capacity influences health financing at national level.

The focus on health system financing is particularly relevant because it underpins progress toward universal health coverage (UHC) and equitable health outcomes. Health financing encompasses the mobilisation, allocation and use of financial resources to support healthcare delivery, including public expenditure, private contributions and out-of-pocket (OOP) payments by households. Effective health system financing can reduce financial barriers to care, improve service delivery and promote health equity. However, financing systems vary widely between countries and their effectiveness may well depend on underlying state capacity, particularly in a context of limited or unevenly distributed fiscal resources.^38^ We employ a considerable range of state capacity indicators, including bureaucratic quality, control of corruption, rule of law, military involvement in politics, government effectiveness, property rights and state fragility, using data for a global sample of 141 countries, including 49 LMICs, for 2000–2020 from multiple well-known datasets. Applying Pooled Ordinary Least Squares with random effects (RE) and fixed effects (FE), our findings indicate an overall positive association between state capacity and health system financing. By integrating multiple measures of state capacity and focusing on health system financing as the outcome, we aim to contribute both to the empirical literature and to policy discussions on strengthening governance to improve health systems worldwide.

The remainder of the article is organised as follows. Section 2 presents the data and variables, Section 3 explains the econometric framework, Section 4 presents the results, Section 5 provides the heterogeneity analyses, Section 6 discusses the findings, and finally, Section 7 draws the conclusions.

### Patient and public involvement

The scope and subject of the paper, based entirely on secondary data and cross-national comparisons, did not explicitly require any involvement of the patient or general public.

## Data and variables

This study uses panel data from 2000 to 2020, drawing on multiple authoritative sources to explore the associations between state capacity and health system financing (2000–2020; data before 2000 are not analysed because most of the measures do not cover earlier years). The primary datasets include the International Country Risk Guide Political Risk Services (ICRG-PRS) (https://www.prsgroup.com/wp-content/uploads/2014/08/icrgmethodology.pdf)(https://www.prsgroup.com/wp-content/uploads/2014/08/prsmethodology.pdf), Varieties of Democracy (V-Dem) (https://www.v-dem.net/static/website/img/refs/methodologyv111.pdf), the Quality of Government Institute (QoG) (https://www.qogdata.pol.gu.se/dataarchive/qog_std_jan22.pdf), Polity V project (https://www.systemicpeace.org/polityproject.html), Global Health Expenditure Database (GHED)(https://apps.who.int/nha/database) and the World Development Indicators (WDI) (https://datatopics.worldbank.org/world-development-indicators/user-guide.html). These sources provide a wide array of indicators to capture diverse dimensions of state capacity, along with key control variables relevant to health financing outcomes.

### State capacity indicators

We conceptualise state capacity through a multidimensional lens, capturing its bureaucratic, legal, fiscal and coercive dimensions. These indicators were selected for their relevance to state capacity’s influence on governance, resource allocation and service delivery, particularly in health systems. The selected indicators include the following, with database sources noted in parentheses. Bureaucratic quality (ICRG-PRS) is assessed on a four-point scale with higher scale values showing higher quality, evaluates the professionalism, autonomy and stability of the civil service. Corruption (ICRG-PRS) is measured on a six-point scale that covers unethical practices such as bribery, favouritism, misuse of public office and patronage, with higher scores representing lower levels of corruption. Thus, the corruption variable reflects the degree of control over corruption.^39^ Rule of Law (V-Dem) is an index scaling from 0 to 1 with higher values indicating a stronger rule of law reflecting judicial independence, adherence to judicial decisions and the effectiveness of law enforcement. Military involvement in politics (ICRG-PRS) evaluates the extent of military influence in political decision-making with the scores ranging from 0 to 6, lower scores signifying greater military participation in politics and vice versa.^40^ As a higher score implicitly reflects stronger civilian control of the government, for clarity, we refer to the *military in politics* variable as *civilian control of government*. Government effectiveness (V-Dem) captures the efficiency and quality of public service delivery, civil service competence and policy implementation, with scores in the range −2.45 to 2.45, a higher score implying better government effectiveness.

Property rights (QoG), scaled from 0 to 1, reflect the strength of legal protections for ownership and contracts. As a key dimension of state capacity, secure property rights signal credible governance and are often used as proxies for rule of law and administrative effectiveness.^41^ Strong property rights reduce arbitrary state action and corruption,^42^ promote economic stability, encourage investment and expand formal economic activity, thereby enhancing fiscal capacity through improved revenue mobilisation.^43^ By limiting rent-seeking and ensuring efficient public resource use, they support better budget execution and help reduce household OOP health spending via increased public investment in health.^44^

The State Fragility Index (QoG) is a composite score assessing the state’s resilience in managing conflict, maintaining stability and delivering public services. Values range from 0 to 25, with higher scores indicating greater fragility.

The state capacity indicators are derived from three sources: the *V-dem dataset*, which collects data on various dimensions of democracy such as clean elections, executive accountability and civil liberties. The dataset draws over 4000 expert ratings whose evaluations are converted into latent variable estimates to correct for coder bias and subjectivity^3 16 23^; the *PRS-ICRG* has 22 components and is widely used to forecast financial, economic and political risk. PRS staff construct each indicator using a standardised methodology on a monthly basis^39 40^, and the *WGI project* is a World Bank research initiative that aggregates data from over 200 countries. Indices are constructed from an aggregation of over 30 data sources, including household surveys, firms and expert assessments,^36^ please see the online supplemental table A17.

In addition to individual indicators, we constructed an aggregate state capacity measure using principal component analysis (PCA) on seven governance dimensions: bureaucratic quality, corruption control, military involvement in politics, state fragility, rule of law, government effectiveness and property rights. PCA was performed on the correlation matrix to normalise scale differences. The first principal component (PC1), with an eigenvalue of 5.13, explained 73% of the total variance and was retained as the composite index, following standard methodological guidance.^45 46^ This component captures the shared institutional variance across indicators and serves as the aggregate state capacity measure in the analysis.

### Outcome variables

This study focuses on two key outcome variables. Government health expenditure (GHE) per capita represents the per capita level of government spending on health services and programmes, expressed in constant US dollars. OOP health expenditure, measured as a share of current health expenditure, serves as the proxy for financial protection, indicating the proportion of health costs directly borne by households at the point of service. While GHE reflects the extent of public investment in health systems, higher OOP levels indicate greater financial burden on households and potential inequities in access to care. Accordingly, we expect stronger state capacity to be positively associated with GHE and negatively associated with OOP health expenditure.

### Control variables

The analyses include a group of relevant control variables. Polity2 scores (Polity V) reflect political regimes, ranging from autocratic to democratic governance. GDP growth and population growth (WDI) captures economic and demographic trends influencing health financing. Urbanisation rate and ageing population (QoG/WDI) represent structural factors that shape health needs and resource distribution. Female educational attainment (QoG) acts as a proxy for societal investments in human capital and its correlation with health outcomes. Current health expenditure as a percentage of GDP (WDI) acts as a control for the scale of health investments across countries.

In this study, we relied entirely on originally reported values from established databases (ICRG-PRS, V-Dem, QoG, Polity V, GHED and WDI). Given the extensive coverage of these datasets—both cross-country and longitudinal—we used an unbalanced panel structure and estimated all regressions using listwise deletion, without applying any statistical imputation. This approach preserves comparability with existing literature on governance and health financing that relies on some of the same secondary datasets.^39 40^

Online supplemental figure F1 visualises the study’s main empirical finding that improvements in state capacity are linked to both increased government investment in health and reduced reliance on OOP payments, supporting the hypothesis that stronger governance underpins more equitable and sustainable health financing systems. Online supplemental figure F4 presents health financing trajectories (2000–2020) for five countries, illustrating trends in OOP spending and GHE per capita across diverse income contexts.

Descriptive statistics (see table 1) for all variables highlight variation across the full sample and within LMICs, underscoring significant disparities in state capacity and health financing measures, which the subsequent analysis investigates.

**Table 1:** Descriptive statistics

	Obs.	Mean	SD	Min.	Max.
Panel A					
Bureaucratic quality	2941	2.19	1.11	0.00	4.00
Control of corruption	2941	2.64	1.19	0.00	6.00
Rule of law	2751	0.58	0.31	0.01	1.00
Civilian control of government	2941	3.81	1.73	0.00	6.00
Government effectiveness	2614	0.08	1.00	−2.44	2.43
Property rights	2751	0.76	0.18	0.03	0.97
State Fragility Index	2558	8.16	6.36	0.00	25.00
Polity2 scores	2495	4.31	6.20	−10.00	10.00
GDP growth	2571	3.46	5.16	−50.34	86.83
Population growth	2625	1.46	1.64	−5.28	19.36
Urban population	2883	61.21	21.36	12.98	100.00
Population 65 years and above	2883	8.33	5.95	0.17	29.58
Female educational attainment	2214	9.49	3.23	1.30	15.60
Current health expenditure % GDP	2536	6.24	2.65	1.55	20.41
Panel B: LMICs					
Bureaucratic quality	1029	1.44	0.80	0.00	3.00
Control of corruption	1029	1.97	0.69	0.00	6.00
Rule of law	945	0.37	0.20	0.01	0.84
Civilian control of government	1029	2.48	1.35	0.00	5.00
Government effectiveness	900	−0.70	0.47	−2.35	0.69
Property rights	945	0.67	0.16	0.12	0.93
State Fragility Index	943	13.89	4.33	3.00	24.00
Polity2 scores	907	2.26	5.32	−9.00	10.00
GDP growth	881	4.32	4.41	−30.15	26.52
Population growth	882	2.18	1.02	−2.88	9.97
Urban population	1041	43.79	17.68	12.98	88.92
Population 65 years and above	1041	3.91	1.52	1.67	10.80
Female educational attainment	796	6.59	2.71	1.30	12.44
Current health expenditure % GDP	872	4.94	2.15	1.55	20.41

GDP, gross domestic product; LMICs, low- and middle-income countries; Obs., observations.

## Econometric framework

We employ ordinary least squares (OLS) regression to examine the associations between different dimensions of state capacity and health system financing in a global sample. OLS provides an intuitive and transparent framework for quantifying the statistical relationships between these measures, making the results accessible to policymakers and applied researchers. Using OLS allows us to assess how variation in each state-capacity indicator is statistically linked to health system financing proxies.

Second, our primary outcome variables—OOP health expenditure and GHE per capita (log-transformed)—are continuous measures appropriately modelled using OLS-based fixed-effects estimation. This approach enables us to examine linear associations between state capacity and health financing while adjusting for a relevant set of economic and demographic covariates.

Nonetheless, including fixed effects and a relevant set of covariates can reduce the influence of some confounding factors; however, this approach does not eliminate all risks of omitted variable bias or endogeneity. Therefore, we explicitly interpret the results as associational rather than causal. Based on these considerations, we estimate the following baseline regression model:


(1) $$HF_{i,c,t}=\alpha +\beta_{i}SC_{i,c,t}+\lambda X_{i}+u_{i}+\epsilon_{i,c,t}$$

where *HF_i,c,t_* is a dependent variable showing the health financing measure *i* of country *c* in year *t*. The variable *SC_i,c,t_* takes into account the state capacity measure *i* of country *c* in time *t*, which includes bureaucratic quality, control of corruption, rule of law, civilian control of government, government effectiveness, property rights and the State Fragility Index. The variable *X_i_* takes the set of covariates, such as Polity2, GDP growth, population growth, current health expenditure, urban population, population ages 65 and above and female educational attainment. Data are in cross-country time series for the period 2000–2020, and estimations are run with both RE and FE in order to capture unobserved time-invariant determinants (*u_i_*). To determine the suitability of the RE versus FE models, we conduct the Hausman test. The Hausman p values indicate that the FE model is preferred, as it allows for controlling time-invariant unobserved heterogeneity.

Online supplemental figures F2 and F3 illustrate that higher state capacity is generally associated with greater public health spending and lower OOP payments, particularly in LMICs. These patterns align with our panel regression findings, highlighting the critical role of state capacity in strengthening financial protection where institutional foundations are weaker.

## Results

The regression results provide an empirical assessment of the relationships outlined in equation 1. To examine the associations between health financing and state capacity, we first conduct univariate regressions for each indicator of state capacity as indicated in columns 1–7, and column 8 includes all the state capacity indicators, in online supplemental tables A1–A4 representing the results of FE and RE models for OOP health expenditures and GHE per capita, respectively.

In addition to the univariate regressions, we conduct multivariate regressions that incorporate relevant control variables in each specification. Moreover, to determine the suitability of the RE versus FE models, the Hausman p values in online supplemental tables A5 and A6 indicate that the FE model is preferred, that is, to allow controlling for time-invariant unobserved heterogeneity. Hence, we have reported the FE model results in the main text, and the RE model results are reported in the online supplemental appendix. Overall, the results indicate statistically significant positive associations between the state capacity measures and health financing outcomes. Our findings are in line with the (limited) existing literature, which, for instance, had shown fiscal capacity to be positively associated with public health expenditure.^38 47^

The data on state capacity indicators are collected from diverse sources and measurement scales to ensure comparability and improve interpretability across measures with different scales, all state capacity indicators are standardised to have a mean of 0 and a SD of 1.

### State capacity and OOP health expenditure

We implement the FE model, with results shown in table 2, the full results are reported in online supplemental table A7, illustrating negative association between state capacity and OOP health expenditures.

**Table 2:** State capacity and health financing (FE model)

	OOP % CHE		GHE per capita (log)	
	(1)	(2)	(3)	(4)
Aggregate SC measure	−0.730		0.175^∗∗∗^	
Bureaucratic quality	(0.536)	−2.434^∗∗^	(0.031)	−0.131^∗^
		(1.226)		(0.070)
Control of corruption		0.312		−0.022
		(0.378)		(0.022)
Rule of law		0.003		0.054
		(0.830)		(0.048)
Civilian control of government		0.979		−0.027
		(0.599)		(0.034)
Government effectiveness		−1.094		0.273^∗∗∗^
		(0.784)		(0.045)
Property rights		−2.214^∗∗^		0.073
		(0.902)		(0.051)
State Fragility Index		0.801		−0.332^∗∗∗^
		(0.763)		(0.044)
Polity2 scores	−0.129	−0.169^∗^	0.003	0.004
	(0.093)	(0.102)	(0.005)	(0.006)
GDP growth (lag)	−0.035	−0.029	0.008^∗∗∗^	0.008^∗∗∗^
	(0.033)	(0.033)	(0.002)	(0.002)
Population growth (lag)	−0.067	−0.043	−0.000	−0.000
	(0.121)	(0.121)	(0.007)	(0.007)
Urban population (lag)	−0.535^∗∗∗^	−0.495^∗∗∗^	0.005	0.002
Population 65 years and above	(0.072) 0.867^∗∗∗^	(0.074) 0.773^∗∗∗^	(0.004) −0.061^∗∗∗^	(0.004) −0.055^∗∗∗^
Female educational attainment (lag)	(0.204) −1.515^∗∗∗^	(0.208) −1.370^∗∗∗^	(0.012) 0.584^∗∗∗^	(0.012) 0.546^∗∗∗^
Current health expenditure % GDP	(0.317) −0.646^∗∗∗^	(0.328) −0.608^∗∗∗^	(0.018)	(0.018)
	(0.156)	(0.159)		
Observations	1757	1757	1757	1757
R^2^ (overall)	0.201	0.211	0.746	0.761

SEs in parentheses. SC measures are standardised to mean 0 and SD 1.

*p*<*0.10, **p*<*0.05, ***p*<*0.01.

CHE, current health expenditure; FE, fixed effects; GDP, gross domestic product; GHE, government health expenditure; OOP, out-of-pocket; SC, state capacity.

Specifically, improvements in bureaucratic quality, higher government effectiveness and stronger property rights are associated with a reduction in OOP health expenditures. The coefficient estimate of the bureaucratic quality variable has a negative sign and is statistically significant at the 5% significance level. The point estimate suggests that a 1 SD increase in the levels of bureaucratic quality is associated with a 2.6 percentage-points decrease in OOP expenditures. Similarly, coefficient estimates of government effectiveness and property rights have negative signs and are statistically significant at 5% and 1% level, respectively. The point estimates suggest that a 1 SD increase in the indicator values of government effectiveness level and property rights are associated with a decrease in OOP % current health expenditure (CHE) of 1.6 percentage-points and 2.8 percentage points.

We first estimate models with each SC indicator separately (online supplemental tables A7 and A8, columns 1–7) to isolate their individual associations with health financing outcomes, a standard approach in the literature.^16^ Recognising state capacity as an interrelated system,^1^ we also estimate joint models including all indicators (columns 9), as well as a PCA-based aggregate state capacity measure (column 8). Results are broadly consistent: the aggregate state capacity measure is negatively but not significantly associated with OOP spending. In the joint model, bureaucratic quality and property rights remain significantly associated with lower OOP, highlighting the role of administrative and institutional strength in reducing household health spending.

We controlled for political regimes using Polity2 scores, which show a negative relationship between the degree of democratisation and OOP health expenditures. Urban population is negatively associated with OOP, while an ageing population increases OOP. Female educational attainment is negatively associated with OOP—higher education levels among women reflecting lower OOP health expenditures. This study includes 1-year lagged covariates (eg, GDP growth, population growth, urban population and female education attainment) to ensure that the explanatory variables temporally precede health outcomes, thereby reducing concerns of simultaneity and reverse causality. This is in keeping with the existing literature in this field (see study by Cingolani *et al*)*.*^16^

The results of the RE model, presented in online supplemental table A5, convey a similar message to the FE model. Specifically, the findings in online supplemental table A5 reaffirm the positive associations between state capacity measures and OOP health expenditures. Furthermore, both the lower-middle-income and upper-middle-income groups exhibit a positive association with higher OOP expenditures compared with the high-income group.

### State capacity and GHE

Results in table 3, the full results are reported in online supplemental table A8, present the association between GHE per capita (in logs) and state capacity measures using FE specifications, while accounting for relevant control variables. Several indicators—including rule of law, government effectiveness and property rights—exhibit positive and statistically significant associations with GHE, suggesting that stronger institutional performance is linked to higher levels of public spending on health. By contrast, greater state fragility is consistently associated with lower GHE per capita.

**Table 3:** State capacity and health financing, LMICs (FE model)

	OOP % CHE		GHE per capita (log)	
	(1)	(2)	(3)	(4)
Aggregate SC measure	−0.233		0.282^∗∗∗^	
Bureaucratic quality	(0.947)	−5.021^∗∗^	(0.045)	0.037
Control of corruption		(2.505) 2.240^∗∗∗^		(0.128) 0.091^∗∗^
		(0.707)		(0.036)
Rule of law		−0.601		0.058
		(1.227)		(0.062)
Civilian control of government		1.896^∗∗^		0.071
Government effectiveness		(0.958) −2.781^∗^		(0.048) 0.287^∗∗∗^
		(1.533)		(0.078)
Property rights		1.652		0.188^∗∗^
		(1.521)		(0.075)
State Fragility Index		6.446^∗∗∗^		−0.081
		(1.447)		(0.073)
Polity2 scores	−0.264^∗∗^	−0.298^∗∗^	0.003	0.008
	(0.134)	(0.146)	(0.007)	(0.007)
GDP growth (lag)	0.035	0.026	0.012^∗∗∗^	0.012^∗∗∗^
	(0.068)	(0.067)	(0.003)	(0.003)
Population growth (lag)	−1.360^∗∗∗^	−1.275^∗∗∗^	−0.007	−0.004
Urban population (lag)	(0.424) −0.707^∗∗∗^	(0.416) −0.722^∗∗∗^	(0.021) 0.017^∗^	(0.021) 0.016^∗^
Population 65 years and above	(0.185) 3.275^∗∗∗^	(0.185) 2.443^∗∗^	(0.009) −0.150^∗∗∗^	(0.009) −0.157^∗∗∗^
	(1.000)	(1.079)	(0.049)	(0.055)
Female educational attainment (lag)	−0.428	1.593^∗^	0.531^∗∗∗^	0.544^∗∗∗^
Current health expenditure % GDP	(0.863) −1.190^∗∗∗^	(0.946) −1.127^∗∗∗^	(0.042)	(0.048)
	(0.263)	(0.266)		
Observations	605	605	605	605
R^2^ (overall)	0.006	0.006	0.625	0.622

SEs in parentheses. SC measures are standardised to mean 0 and SD 1.

*p*<*0.10, **p*<*0.05, ***p*<*0.01.

CHE, current health expenditure; FE, fixed effects; GDP, gross domestic product; GHE, government health expenditure; LMICs, low- and middle-income countries; OOP, out-of-pocket; SC, state capacity.

The magnitude of these coefficients indicates that a 1 SD increase in rule of law, government effectiveness or property rights is associated with increases in GHE per capita of approximately 13%–31%. Conversely, a 1 SD rise in the State Fragility Index corresponds to a reduction of around 32% in GHE per capita. The *aggregate state capacity measure*, constructed via PCA (column 8), is positively and significantly associated with GHE per capita, reinforcing the broader conclusion that stronger institutional capacity supports higher public investment in health. A 1 SD rise in the aggregate state capacity corresponds to increase of around 17.5% in GHE per capita. As expected, the State Fragility Index retains a negative and significant association, indicating that more fragile institutional environments are linked with lower public spending on health. These coefficient plots confirm that stronger state capacity (property rights, bureaucratic quality) lowers the OOP expenditures.

The negative and significant coefficient for bureaucratic quality in column 9 arises from correlation among state capacity indicators.^1^ When correlated indicators are entered together, the shared variance is redistributed, causing a sign reversal that does not imply a true negative relationship. The PCA-based state capacity measure avoids this issue and consistently shows a positive association with GHE, as supported by the correlation matrix in online supplemental table A14.

Among the covariates, democratic governance (Polity2 scores), GDP growth and female educational attainment are positively associated with GHE per capita, whereas a higher share of the population aged 65 years and above is associated with lower levels of public health spending. The RE results in online supplemental table A6 are broadly consistent with these findings, with civilian control of government additionally showing a significant positive association with GHE per capita.

Online supplemental figure F5 plots coefficient estimates from the joint fixed-effects models, showing that stronger institutional features—particularly property rights and bureaucratic quality—are associated with lower OOP spending, while government effectiveness is positively linked to higher public health expenditure. Conversely, greater state fragility is significantly associated with reduced government health spending, underscoring the importance of institutional strength for health financing.

## Heterogeneity test

We conducted subgroup analyses to examine whether the associations between state capacity and health financing outcomes differ across countries by income classification. table 3, full results are reported in online supplemental tables A9 and A10, presents the fixed-effects regression results for the LMIC subsample. Compared with the full-sample estimates reported in table 2, full results are reported online supplemental tables A7 and A8, the coefficients for the LMICs are larger in magnitude yet remain directionally consistent with the main findings. Improvements in state capacity indicators are generally associated with lower OOP health spending and higher government health expenditure per capita. The higher coefficients observed among LMICs suggest that institutional capacity may play a particularly important role in shaping health financing performance in LMICs.

An exception is observed for the *control of corruption* and *State Fragility Index* in the OOP regressions, where the coefficients turn positive and statistically significant among LMICs. This finding may suggest that, in contexts with weak institutional oversight and limited fiscal discipline, greater emphasis on accountability and anti-corruption reforms can initially expose inefficiencies or shift resources away from service delivery, thereby increasing households’ reliance on private spending. Similar dynamics have been noted in prior studies, where anti-corruption initiatives may paradoxically raise corruption per unit of public spending in the short term, leading to higher OOP health expenditures.^48^

Online supplemental figure F6 presents the associations between state capacity indicators and the two health-financing outcomes for the LMIC subsample. The patterns broadly mirror the full-sample results but are stronger in magnitude.

To assess variation across institutional contexts, we created terciles of the aggregate SC index—low, medium and high. Fixed-effects regression results (online supplemental table A13) show that countries in the middle tercile exhibit significantly lower OOP spending and higher GHE per capita compared with the lowest tercile. In contrast, countries in the highest state capacity group do not differ significantly from the lowest group, likely due to limited within-country variation among high-capacity states. These findings suggest a non-linear association: the greatest gains in public health financing and financial protection occur when countries improve from low to moderate state capacity levels. As expected, control variables such as stronger democracy, higher GDP growth, female education and urbanisation are positively associated with favourable health financing outcomes.

Given conceptual overlap and correlation among state capacity indicators, we employed two strategies to address potential multicollinearity. First, we estimated joint models including all indicators simultaneously (tables2  3, and in online supplemental tables A7–A10, column 9), enabling assessment of each dimension’s contribution while controlling for others. Second, we constructed a PCA-based aggregate state capacity index that condenses correlated indicators into a single standardised measure of shared institutional variance. We re-estimated our main models using this index (online supplemental tables A7–A10, column 8). Results from both approaches are consistent with baseline findings: stronger state capacity is associated with lower OOP spending and higher GHE per capita. These robustness checks reinforce the validity of our results and suggest they are not artefacts of collinearity among the governance measures.

### Reverse causality

As our primary outcomes include GHE per capita, which may raise concerns about potential reverse causality—that higher public health spending could in turn strengthen state capacity. To address this, we estimated regression models using lagged state capacity indicators to assess whether improvements in governance precede changes in health spending rather than the other way. One of the advantages of using previous values of the state capacity indicators is that it minimises the chances of reverse causality between GHE per capita and state capacity indicators, as government health spending of the current time cannot affect the past state capacity.^49 50^ Results are presented in online supplemental tables A11 and A12. These robustness checks reinforce our main findings and suggest that the observed associations are unlikely to be driven by reverse causality.

## Discussion

This study moves beyond traditional macro-fiscal analyses by examining the political-economy determinants of public health expenditure across multiple dimensions of state capacity. In doing so, it builds on a growing body of work showing that public spending on health is shaped not only by economic conditions but also by political institutions, governance structures and policy choices.^51^,^53^ The findings therefore contribute to a deeper empirical understanding of the political economy factors that influence governments’ commitment to, and investment in, national health systems.

We provide a comprehensive analysis of the relationship between various measures of state capacity and health system financing across countries. We consider state capacity as a combination of several dimensional indicators, which include bureaucratic quality, control of corruption, rule of law, military in politics, government effectiveness and state fragility. Overall, the results consistently show a positive association between state capacity and health financing outcomes and offer several key insights.

Our primary outcomes of interest—OOP health expenditure as a percentage of CHE, and GHE per capita—are widely used indicators in global health financing studies.^54^,^57^ Public health spending, measured on a per capita basis, serves as a key indicator of a state’s commitment to its health system. Sustained growth in public health expenditure is considered essential for advancing progress toward UHC.^58 59^ Our study demonstrates a positive relationship between health financing and state capacity, where stronger state capacity parameters are associated with lower OOP and higher GHE. Enhanced state capacity improves access to and the quality of public health facilities, thereby reducing the financial burden on individuals by incurring OOP expenditure in seeking care elsewhere, mostly in private facilities. Higher levels of state capacity, by expanding fiscal and administrative capacities, are also likely to entail higher per capita public spending on publicly-provided services reinforcing the virtuous cycle of strengthened state capacity and improved health financing outcomes.^60 61^

This study conceptualises state capacity as a multidimensional construct, recognising that governments exercise authority and deliver services through several interrelated institutional functions. Previous work has highlighted different components of this concept. For example, Cingolani *et al*^16^ emphasise bureaucratic autonomy as a critical dimension, showing that stronger and more independent bureaucracies are associated with improved health outcomes, including reductions in child mortality and tuberculosis prevalence. Other studies use broader measures of institutional quality as proxies for state capacity and similarly find beneficial associations—for instance, lower child mortality in settings with stronger governance^62^ and improvements in the quality of public health service delivery.^63^ Together, this evidence underscores the importance of considering multiple dimensions of state capacity when examining its implications for health system performance.

Along similar lines, our findings also indicate that higher bureaucratic quality is associated with lower OOP health expenditures, underscoring the critical role of administrative capacity in shaping health system financing. Strengthening administrative quality contributes to democratic and meritocratic governance, infrastructure development, improved public education and enhanced access to and quality of public healthcare services—all of which help alleviate the financial burden of OOP expenses on individuals by stimulating better performance of public systems and encouraging efficient use of available resources. However, the relationship between bureaucratic quality and GHE per capita is not statistically significant. A possible explanation is that health budget allocations are often shaped by political priorities, whereas the efficient utilisation of allocated funds is more closely tied to bureaucratic effectiveness.

Studies have also used control of corruption as a proxy for state capacity, finding that higher corruption is associated with lower education and health spending, as well as poorer health outcomes.^39 64 65^ Corruption generally weakens healthcare delivery and increases OOP health expenditures for patients.^66^ In the case of LMICs, our findings indicate a positive and statistically significant association between control of corruption and GHE per capita, suggesting that effective control, and accordingly, lower levels of corruption unlock higher levels of public spending on health. However, its impact on other aspects of health financing is not straightforward. While a positive relationship between control of corruption and health financing is the dominant result, there are cases where anti-corruption efforts are associated with worsening health indicators, for example, in the case of Brazil’s anti-corruption programme.^48^

An exception is observed for the control of corruption and state fragility indicators in the OOP regressions, where the coefficients turn positive and statistically significant among LMICs. This pattern may indicate that, in contexts with weak institutional oversight and budgetary discipline, greater emphasis on accountability reforms can initially expose inefficiencies or reallocate funds away from service delivery, thereby increasing private health spending. Similar short-term effects have been reported in prior literature, where anti-corruption initiatives paradoxically raise corruption per unit of public spending, leading to higher OOP burdens.^48^

Our findings also show that, particularly in LMICs, control of corruption is positively associated with OOP health expenditures. One possible explanation is that corruption can create incentives for officials to deliver services, and curbing it may inadvertently harm downstream outcomes. This effect could be mediated by accountability on public spending, leading to higher corruption per dollar spent, ultimately increasing individuals’ OOP health expenditures. For instance, politicians may strategically allocate health budgets to benefit politically connected firms or allies through lucrative contracts. (For further details on corruption and health outcomes, see studies by Lichand *et al*,^48^ Mæstad and Mwisongo^67^ and Liang and Mirelman.^68^)

Research has shown a positive association between adherence to the rule of law and health outcomes.^69^ The legal and justice sector plays a crucial role in addressing health challenges, including the performance of UHC-oriented systems.^70^ Our findings indicate that stronger adherence to the rule of law is consistently associated with better health financing outcomes, characterised by both lower OOP expenditures and higher GHE.

Studies have examined the impact of military involvement in politics on health outcomes and government health expenditure. Shin^71^ showed that greater influence of the military in politics has a negative effect on government health spending. Similarly, Elgin *et al*^72^ and Ikegami and Wang^35^ suggested that military expenditure crowds out health spending and is negatively associated with health outcomes. Our findings indicate that, particularly in the context of LMICs, reducing military involvement in politics could significantly increase per capita government health expenditure. These results align with the existing literature (see a study by Shin).^71^

For governments, particularly in LMICs, a key challenge is to increase health budgets, enhance the quality of health services and ensure equity in healthcare. Previous studies have highlighted that higher government efficiency can significantly improve both health spending^73^ and health outcomes.^36 74^ Similarly, our findings also indicate that government effectiveness has a significant positive impact on health financing, with improved governance leading to a reduction in OOP expenditures and an increase in GHE per capita.

Stable property rights encourage long-term investment in health infrastructure.^37^ Our findings show that better protection of property rights is linked to improved health financing indicators and that higher levels of state fragility are associated with lower levels of health financing, such as higher OOP expenditure and lower GHE per capita.

Our study also considers various contextual correlates of health financing. For example, the relationship between state capacity and health financing varies across political regimes^36 75^ and across income groups.^39^ Accordingly, we controlled for political regimes using Polity2 scores that are showing positive associations between democratisation and health financing outcomes. We also controlled for country-income groups with lower-middle and upper-middle-income countries showing different patterns compared with high-income countries.

Our findings suggest several potential pathways through which stronger state capacity can shape health financing performance. Improvements in bureaucratic quality and rule-based governance are likely to enhance administrative efficiency, strengthen procurement and financial oversight systems and reduce leakages in public spending.^17 76^ These mechanisms may contribute to lower reliance on OOP payments, thereby improving financial protection.

The analysis also indicates that while bureaucratic quality has a robust negative association with OOP spending, its effect on GHE per capita is weaker. This may reflect the administrative rather than fiscal nature of bureaucratic improvements—enhancing efficiency and accountability in spending rather than expanding the overall fiscal envelope.^16 77^ By contrast, dimensions such as government effectiveness, rule of law and property rights are more directly linked to resource mobilisation and budget execution, explaining their stronger association with GHE.^78^,^80^

These results have implications for the sequencing of governance reforms. Strengthening administrative and bureaucratic systems may be a necessary precursor to fiscal reforms in health financing. Improvements in bureaucratic quality, regulatory oversight and procurement systems can enhance spending efficiency and accountability, ensuring that additional public resources are used effectively once fiscal space expands.^17 76 81^ In this sense, administrative capacity acts as the foundation on which fiscal reforms can generate sustainable and equitable health financing outcomes.

Despite its valuable insights, this study has some limitations. First, endogeneity could be a potential issue. While FE models control for unobserved heterogeneity, they do not fully address potential bias from unobserved time-varying confounding. Future research could add value by identifying and using instrumental variable approaches or other causal inference techniques. Second, measuring state capacity remains inherently complex, and while this study incorporates widely used governance indicators, it does not account for informal institutions or political fragmentation, potentially omitting key determinants of health financing. Additionally, reliance on subjective governance indices (eg, ICRG, V-Dem, QoG) may introduce reporting biases. Third, data coverage limitations affect generalisability, as gaps in state capacity indicators across countries may influence results, and national-level data may not fully capture subnational disparities in governance and healthcare access. Fourth, a further limitation concerns the temporal alignment between governance measures and health spending outcomes. To improve plausibility, we estimated models using 1-year lagged state capacity indicators, linking institutional conditions to subsequent changes in health expenditure (online supplemental tables A11 and A12). This approach mitigates simultaneity concerns but cannot fully capture delayed or gradual effects of governance reforms, as most governance indicators are annual and reflect slow-moving institutional change. The findings should therefore be interpreted as robust associations rather than strict causal effects. Addressing these limitations through causal methods and regional analyses would enhance the robustness and applicability of the findings.

## Conclusion

This study provides robust empirical evidence that state capacity—understood across administrative, legal and institutional dimensions—plays a critical role in shaping national health financing patterns. Using a global panel dataset of 141 countries from 2000 to 2020, we demonstrate that higher levels of bureaucratic quality, rule of law, government effectiveness and property rights (particularly in univariate models) are significantly associated with increased government health expenditure and lower OOP spending by households. Conversely, greater state fragility is linked to underinvestment in public health and higher financial burdens on individuals.

The findings underscore that achieving UHC and ensuring equitable access to healthcare are not solely fiscal challenges—they are, fundamentally, questions of governance. Strengthening the capacity of state institutions to allocate and manage resources, deliver public services and uphold legal accountability is central to building resilient and inclusive health systems.

From a policy perspective, particularly in LMICs, these results emphasise the need for governance reforms aimed at strengthening institutional capacity to ensure sustainable and equitable health financing. Governments should consider prioritising the development of bureaucratic professionalism, reinforcing anti-corruption measures and fostering legal stability to improve public health investment. Additionally, mitigating state fragility through improved political stability and institutional resilience can play a crucial role in fostering efficient resource allocation within the health sector.

Future research should explore subnational variation in state capacity and health financing, examine the role of decentralisation and apply causal methods to further unpack the mechanisms linking governance quality to health financing. Strengthening state capacity is not a peripheral concern—it is a foundational requirement for sustainable, equitable health systems.

## Supplementary material

10.1136/bmjgh-2025-020101 online supplemental file 1

10.1136/bmjgh-2025-020101 online supplemental file 2

## Data Availability

Data are available in a public, open access repository. Data are available upon reasonable request. Data may be obtained from a third party and are not publicly available.
