# Supplementary material for: State capacity and health system financing: a cross-country analysis
Source: BMJ Glob Health. 2026 Mar 24;11(3):e020101. doi: 10.1136/bmjgh-2025-020101 (PMC13034253; doi:10.1136/bmjgh-2025-020101)
Supplement: online supplemental file 1 [file bmjgh-11-3-s001.docx]

# Appendix A

[Figure F1](#_bookmark0) illustrates the cross-sectional relationship between the aggregate state capacity measure and two key indicators of health system financing for the period 2000–2020. The upper panel shows a clear positive association between the aggregate state capacity measure based on PCA and GHE per capita (in logarithmic terms). Countries with stronger institutional capacity —reflecting higher bureaucratic quality, rule of law, and governance effectiveness— tend to allocate more public resources to health, consistent with the regression results (Table [A8](#_bookmark7)).

In contrast, the lower panel depicts a negative relationship between aggregate state capacity and OOP expenditure as a share of current health expenditure. Higher state capacity is associated with lower household financial contributions to health care, indicating stronger public financing mechanisms and improved financial protection. The dispersion of data points is wider among countries with weaker state capacity, suggesting greater heterogeneity in health financing performance within this group

Figures [F2](#_bookmark1) and [F3](#_bookmark2) depict bivariate associations between aggregate state capacity and health financing outcomes —GHE per capita and OOP spending as a share of current health expenditure— for six representative countries. Among low- and middle-income countries such as India, Brazil, and Vietnam, higher state capacity is generally associated with increased public health spending and reduced reliance on OOP payments, reflecting progress toward stronger financial protection. In contrast, for high-income countries such as Sweden and Finland with consistently high GHE and low OOP levels, there is a limited variation across the state capacity spectrum, indicating institutional maturity and fiscal stability. These cross-country trajectories are consistent with the panel regression results that follow, suggesting that improvements in state capacity are particularly consequential for health financing in lower-capacity settings.

In the appendix Figure [F4](#_bookmark3) presents a set of time-series trajectory plots illustrating the evolution of health financing outcomes in five representative countries —India, Brazil, Egypt, Vietnam, and Sweden— selected to capture variation between LMICs and high- income countries (HICs). The figure depicts trends in both OOP expenditure and GHE per capita over the period 2000–2020.

## Figure F1: State capacity and health expenditure (2000–2020)

*
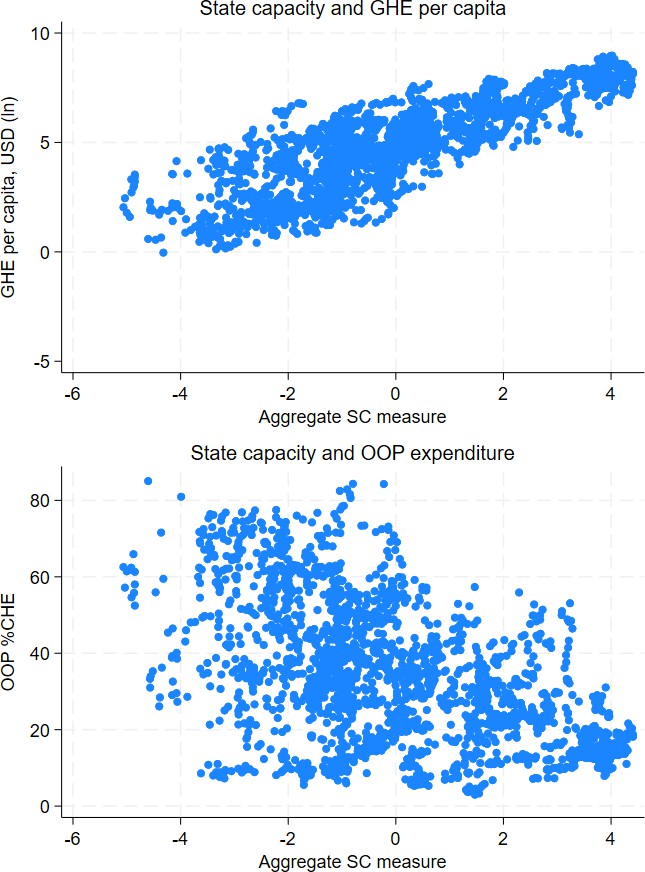
*

*Note:* Author’s calculations based on data for 2000–2020. The upper panel shows that higher state capacity is associated with greater government health expenditure per capita, while the lower panel indicates that stronger state capacity corresponds to lower out-of-pocket spending as a share of current health expenditure.

## Figure F2: OOP expenditure and State capacity (2000–2020)

*
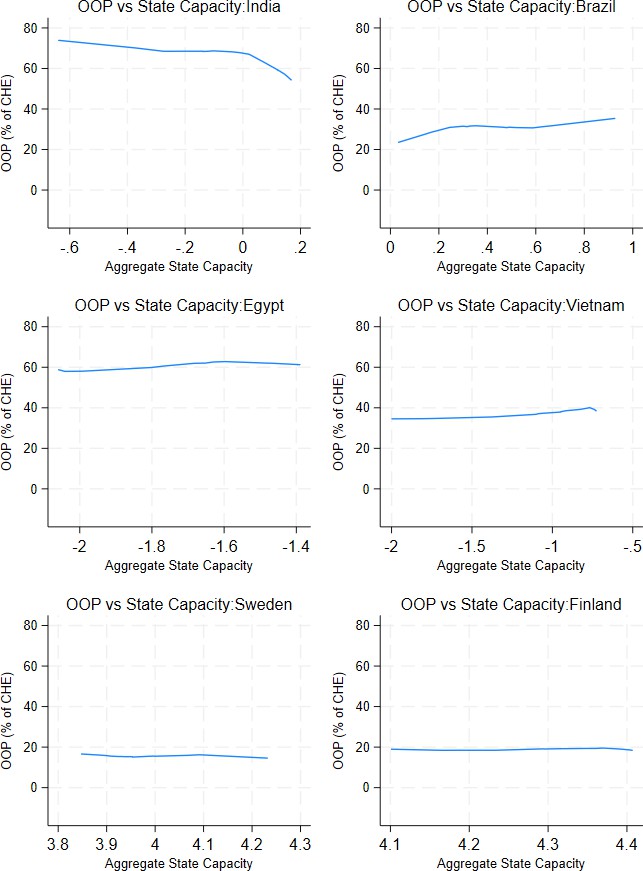
*

*Note:* Each panel illustrates the relationship between the aggregate state-capacity and OOP expenditure (% of CHE) for six representative countries—India, Brazil, Egypt, Vietnam, Sweden, and Finland. A clear inverse relationship is observed in most low- and middle-income countries, indicating that stronger State capacity is associated with reduced OOP expenditure, whereas high-income countries show consistently low OOP levels with minimal variation across state- capacity values

## Figure F3: GHE per capita and State capacity (2000–2020)

*
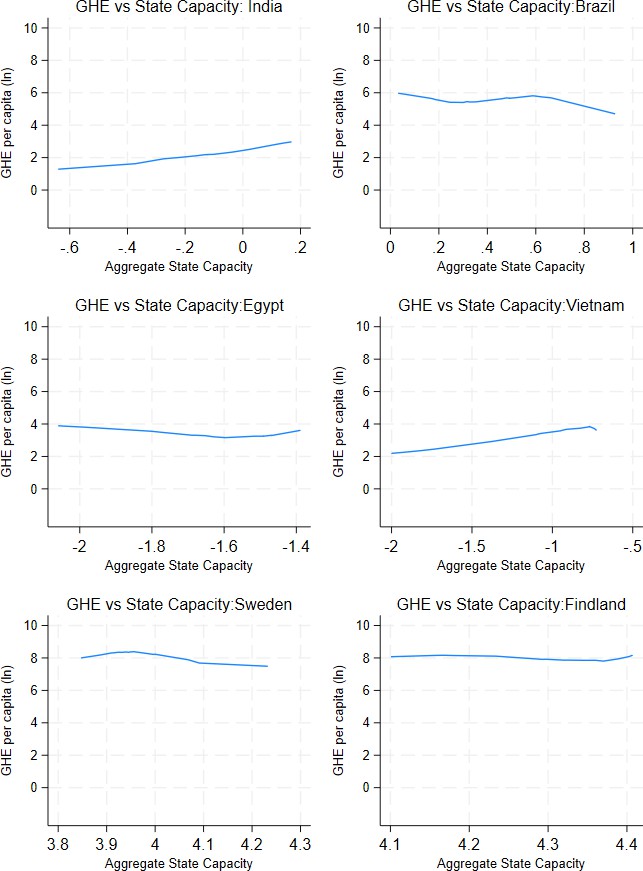
*

*Note:* Each panel illustrates the relationship between the aggregate state-capacity and government health expenditure per capita (log-transformed). In countries such as India, Vietnam, and Brazil, higher state capacity corresponds to greater GHE, whereas Sweden and Finland show stable high levels of GHE, reflecting mature aggregate state capacity.

## Figure F4: State capacity and health expenditure (2000–2020)

*
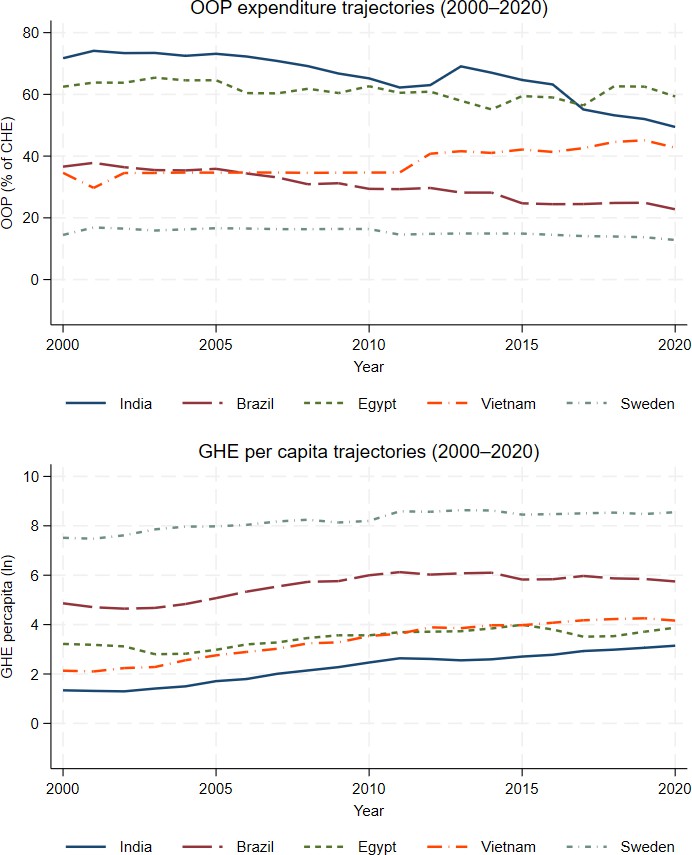
*

*Note:* Author’s calculations based on the randomly selected countries data for 2000–2020. The upper panel shows out-of-pocket (OOP) health spending as a percentage of current health expenditure (CHE), while the lower panel shows government health expenditure (GHE) per capita (in natural log). The five countries (India, Brazil, Egypt, Vietnam, and Sweden) illustrate a mix of low-, middle-, and high-income contexts, highlighting cross-country variation in financing trajectories. Both indicators show overall declines in OOP spending and gradual increases in GHE percapita across most LMICs during the two-decade period.

[Figure F5](#_bookmark4) plots the coefficient estimates with 95% confidence intervals (CIs) from the joint fixed-effects models (column 9) of Table ([A7](#_bookmark6)) (OOP % CHE) and Table ([A8](#_bookmark7)) (GHE per capita). Each dot is the coefficient for one standardized state-capacity indicator; capped lines are 95% CIs, models include the full set of covariates. For OOP expenditures, the coefficient plot shows that stronger state capacity, particularly property rights and bureaucratic quality, is associated with lower reliance on household OOP payments. These patterns reflect the role of institutional quality in supporting more predictable, coordinated, and publicly financed health systems.

For GHE per capita, government effectiveness displays a positive and statistically significant association, suggesting that countries with stronger administrative performance tend to allocate and execute higher levels of public spending on health. Conversely, the state fragility index shows a negative and significant relationship, indicating that more fragile institutional environments are systematically linked with lower public spending on health.

[Figure F6](#_bookmark5) presents the associations between state capacity indicators and the two health-financing outcomes for the LMIC sub-sample. The patterns broadly mirror the full-sample results but are stronger in magnitude.

## Figure F5: Coefficient plots: State capacity and health financing (FE models)

*
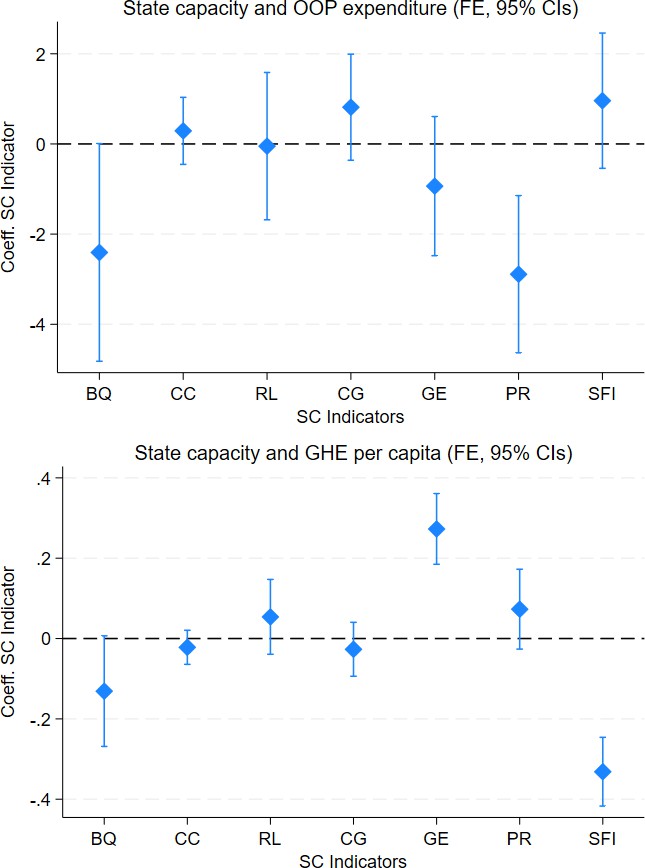
*

*Note:* Author’s calculation based on panel data from 2000–2020. The figure presents coefficient estimates with 95% confidence intervals from fixed-effects regressions examining the association between state capacity indicators and (i) out-of-pocket health expenditure (% of current health expenditure) and (ii) government health expenditure per capita. State capacity indicators include Bureaucratic Quality (BQ), Control of Corruption (CC), Rule of Law (RL), Civilian Control of Government (CG), Government Effectiveness (GE), Property Rights (PR), and State Fragility Index (SFI). All models control for GDP growth, population growth, urbanization, ageing population, democracy scores, female educational attainment, and current health expenditure as a share of GDP (for the model OOPs outcome variable). 95% confidence intervals are shown by capped lines.

## Figure F6: Coefficient plots, LMICs: State capacity and health financing (FE models)

*
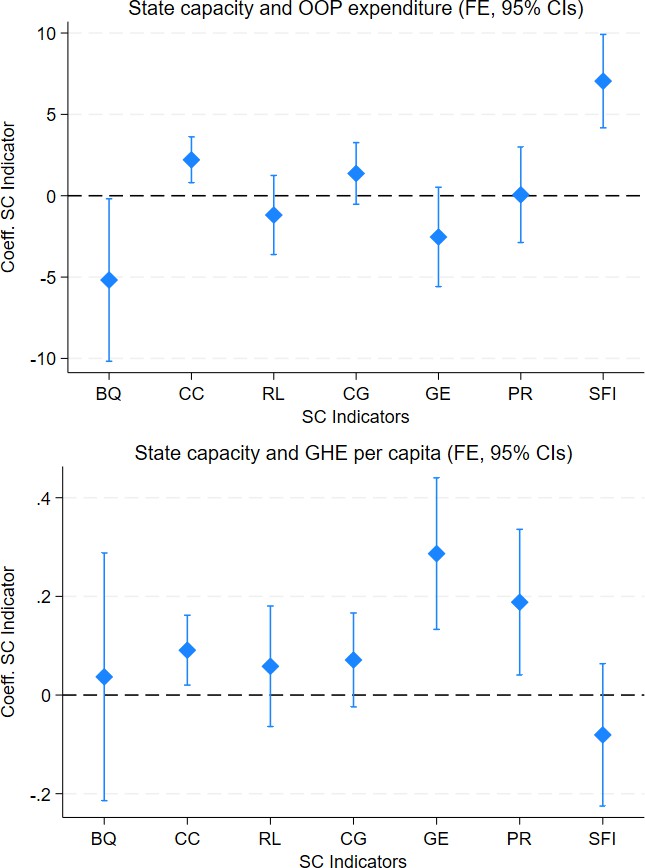
*

*Note:* Author’s calculation based on panel data for LMICs (2000–2020). The figure presents coefficient estimates with 95% confidence intervals from fixed-effects regressions examining the association between state capacity indicators and (i) out-of-pocket health expenditure (% of current health expenditure) and (ii) government health expenditure per capita. State capacity indicators include Bureaucratic Quality (BQ), Control of Corruption (CC), Rule of Law (RL), Civilian Control of Government (CG), Government Effectiveness (GE), Property Rights (PR), and State Fragility Index (SFI). All models control for GDP growth, population growth, urbanization, ageing population, democracy scores, female educational attainment, and current health expenditure as a share of GDP (for the model OOPs outcome variable). 95% confidence intervals are shown by capped lines.

## Table A1: State capacity and Out-of-pocket health expenditure (RE model)

|  | (1) | (2) | (3) | (4) | (5) | (6) | (7) | (8) |
| --- | --- | --- | --- | --- | --- | --- | --- | --- |
| OOP %CHE |  |  |  |  |  |  |  |  |
| Bureaucratic quality | -0.191^∗∗∗^ |  |  |  |  |  |  | -0.077^∗∗^ |
| Control of corruption | (0.026) | -0.060^∗∗∗^ |  |  |  |  |  | (0.032)  -0.051^∗∗∗^ |
| Rule of law |  | (0.011) | -0.026 |  |  |  |  | (0.012)  0.047^∗∗^ |
| Civilian control of govt. |  |  | (0.018) | -0.004 |  |  |  | (0.022)  0.070^∗∗∗^ |
|  |  |  |  | (0.016) |  |  |  | (0.018) |
| Govt. effectiveness |  |  |  |  | -0.100^∗∗∗^ |  |  | -0.005 |
| Property rights |  |  |  |  | (0.020) | -0.148^∗∗∗^ |  | (0.024)  -0.095^∗∗∗^ |
| State fragility Index |  |  |  |  |  | (0.020) | 0.243^∗∗∗^ | (0.025)  0.217^∗∗∗^ |
|  |  |  |  |  |  |  | (0.017) | (0.019) |
| *Observations* | 2745 | 2745 | 2556 | 2745 | 2435 | 2556 | 2421 | 2132 |
| *R*^2^(*overall*) | 0.195 | 0.199 | 0.239 | 0.229 | 0.233 | 0.037 | 0.257 | 0.163 |

Standard errors in parentheses. SC measure are standardized to mean 0 and SD 1

^∗^ *p <* 0*.*10, ^∗∗^ *p <* 0*.*05, ^∗∗∗^ *p <* 0*.*01

## Table A2: State capacity and Out-of-pocket health expenditure (FE model)

|  | (1) | (2) | (3) | (4) | (5) | (6) | (7) | (8) |
| --- | --- | --- | --- | --- | --- | --- | --- | --- |
| OOP %CHE |  |  |  |  |  |  |  |  |
| Bureaucratic quality | -0.151^∗∗∗^ |  |  |  |  |  |  | -0.063^∗^ |
| Control of corruption | (0.031) | -0.047^∗∗∗^ |  |  |  |  |  | (0.036)  -0.048^∗∗∗^ |
| Rule of law |  | (0.011) | 0.026 |  |  |  |  | (0.012)  0.076^∗∗∗^ |
| Civilian control of govt. |  |  | (0.020) | 0.034^∗∗^ |  |  |  | (0.022)  0.087^∗∗∗^ |
|  |  |  |  | (0.016) |  |  |  | (0.019) |
| Govt. effectiveness |  |  |  |  | -0.055^∗∗^ |  |  | 0.013 |
| Property rights |  |  |  |  | (0.022) | -0.148^∗∗∗^ |  | (0.024)  -0.132^∗∗∗^ |
| State fragility Index |  |  |  |  |  | (0.021) | 0.231^∗∗∗^ | (0.026)  0.211^∗∗∗^ |
|  |  |  |  |  |  |  | (0.018) | (0.020) |
| *Observations* | 2745 | 2745 | 2556 | 2745 | 2435 | 2556 | 2421 | 2132 |
| *R*^2^(*overall*) | 0.195 | 0.199 | 0.239 | 0.229 | 0.233 | 0.037 | 0.257 | 0.103 |

Standard errors in parentheses. SC measure are standardized to mean 0 and SD 1

^∗^ *p <* 0*.*10, ^∗∗^ *p <* 0*.*05, ^∗∗∗^ *p <* 0*.*01

## Table A3: State capacity and Govt. Heath expenditure per capita (RE model)

|  | (1) | (2) | (3) | (4) | (5) | (6) | (7) | (8) |
| --- | --- | --- | --- | --- | --- | --- | --- | --- |
| GHE per capita |  |  |  |  |  |  |  |  |
| Bureaucratic quality | 0.884^∗∗∗^ |  |  |  |  |  |  | 0.213^∗∗∗^ |
| Control of corruption | (0.071) | 0.168^∗∗∗^ |  |  |  |  |  | (0.069)  0.110^∗∗∗^ |
|  |  | (0.031) |  |  |  |  |  | (0.028) |
| Rule of law |  |  | 0.431^∗∗∗^ |  |  |  |  | -0.006 |
| Civilian control of govt | . |  | (0.052) | 0.222^∗∗∗^ |  |  |  | (0.049)  -0.196^∗∗∗^ |
| Govt. effectiveness |  |  |  | (0.044) | 0.950^∗∗∗^ |  |  | (0.041)  0.383^∗∗∗^ |
| Property rights |  |  |  |  | (0.051) | 0.651^∗∗∗^ |  | (0.054)  0.231^∗∗∗^ |
| State fragility Index |  |  |  |  |  | (0.056) | -1.492^∗∗∗^ | (0.055)  -1.295^∗∗∗^ |
|  |  |  |  |  |  |  | (0.040) | (0.045) |
| *Observations* | 2745 | 2745 | 2556 | 2745 | 2435 | 2556 | 2421 | 2132 |
| *R*^2^(*overall*) | 0.569 | 0.448 | 0.482 | 0.493 | 0.713 | 0.264 | 0.774 | 0.801 |

Standard errors in parentheses. SC measure are standardized to mean 0 and SD 1

^∗^ *p <* 0*.*10, ^∗∗^ *p <* 0*.*05, ^∗∗∗^ *p <* 0*.*01

## Table A4: State capacity and Govt. Heath expenditure per capita (FE model)

|  | (1) | (2) | (3) | (4) | (5) | (6) | (7) | (8) |
| --- | --- | --- | --- | --- | --- | --- | --- | --- |
| GHE per capita |  |  |  |  |  |  |  |  |
| Bureaucratic quality | 0.477^∗∗∗^ |  |  |  |  |  |  | 0.041 |
| Control of corruption | (0.085) | 0.095^∗∗∗^ |  |  |  |  |  | (0.083)  0.099^∗∗∗^ |
|  |  | (0.031) |  |  |  |  |  | (0.028) |
| Rule of law |  |  | 0.244^∗∗∗^ |  |  |  |  | -0.039 |
| Civilian control of govt | . |  | (0.055) | 0.053 |  |  |  | (0.052)  -0.241^∗∗∗^ |
| Govt. effectiveness |  |  |  | (0.045) | 0.607^∗∗∗^ |  |  | (0.043)  0.309^∗∗∗^ |
| Property rights |  |  |  |  | (0.058) | 0.590^∗∗∗^ |  | (0.057)  0.319^∗∗∗^ |
| State fragility Index |  |  |  |  |  | (0.058) | -1.364^∗∗∗^ | (0.061)  -1.255^∗∗∗^ |
|  |  |  |  |  |  |  | (0.044) | (0.046) |
| *Observations* | 2745 | 2745 | 2556 | 2745 | 2435 | 2556 | 2421 | 2132 |
| *R*^2^(*overall*) | 0.569 | 0.448 | 0.482 | 0.493 | 0.713 | 0.264 | 0.774 | 0.779 |

Standard errors in parentheses. SC measure are standardized to mean 0 and SD 1

^∗^ *p <* 0*.*10, ^∗∗^ *p <* 0*.*05, ^∗∗∗^ *p <* 0*.*01

## Table A5: State capacity and Out-of-pocket health expenditure (RE model)

|  | (1) | (2) | (3) | (4) | (5) | (6) | (7) | (8) |
| --- | --- | --- | --- | --- | --- | --- | --- | --- |
| OOP %CHE |  |  |  |  |  |  |  |  |
| Bureaucratic quality | -2.788^***^ |  |  |  |  |  |  | -2.331^**^ |
|  | (0.986) |  |  |  |  |  |  | (1.086) |
| Control of corruption |  | 0.262 |  |  |  |  |  | 0.285 |
|  |  | (0.346) |  |  |  |  |  | (0.379) |
| Rule of law |  |  | -1.488^*^ |  |  |  |  | -0.714 |
|  |  |  | (0.771) |  |  |  |  | (0.800) |
| Civilian control of govt. |  |  |  | 0.008 |  |  |  | 0.609 |
|  |  |  |  | (0.520) |  |  |  | (0.585) |
| Govt. effectiveness |  |  |  |  | -1.707^**^ |  |  | -0.952 |
|  |  |  |  |  | (0.722) |  |  | (0.775) |
| Property rights |  |  |  |  |  | -2.533^***^ |  | -1.516^*^ |
|  |  |  |  |  |  | (0.771) |  | (0.830) |
| State fragility Index |  |  |  |  |  |  | 1.779^***^ | 1.456^*^ |
|  |  |  |  |  |  |  | (0.679) | (0.748) |
| GDP growth (lag) | -0.046 | -0.047 | -0.048 | -0.047 | -0.038 | -0.039 | -0.041 | -0.031 |
|  | (0.032) | (0.033) | (0.033) | (0.033) | (0.033) | (0.033) | (0.032) | (0.033) |
| Population growth (lag) | -0.077 | -0.074 | -0.064 | -0.077 | -0.073 | -0.051 | -0.074 | -0.054 |
|  | (0.121) | (0.122) | (0.123) | (0.122) | (0.121) | (0.122) | (0.121) | (0.122) |

Urban Population (lag) -0.349^***^ -0.357^***^ -0.344^***^ -0.346^***^ -0.351^***^ -0.348^***^ -0.328^***^ -0.303^***^ (0.055) (0.055) (0.055) (0.055) (0.058) (0.056) (0.055) (0.057)

Pop 65y and above 0.763^***^ 0.756^***^ 0.742^***^ 0.750^***^ 0.779^***^ 0.723^***^ 0.725^***^ 0.721^***^ (0.172) (0.172) (0.170) (0.171) (0.177) (0.172) (0.170) (0.177)

Polity2scores -0.175^**^ -0.192^**^ -0.115 -0.192^**^ -0.210^**^ -0.137 -0.166^**^ -0.113

(0.080) (0.080) (0.093) (0.082) (0.084) (0.084) (0.081) (0.097)

Female edu attain (lag) -1.918^***^ -1.916^***^ -1.918^***^ -1.930^***^ -1.882^***^ -1.836^***^ -1.728^***^ -1.704^***^ (0.262) (0.262) (0.259) (0.261) (0.272) (0.262) (0.270) (0.284)

Current health exp%GDP-0.610^***^ -0.609^***^ -0.563^***^ -0.607^***^ -0.642^***^ -0.494^***^ -0.569^***^ -0.583^***^ (0.146) (0.146) (0.149) (0.147) (0.152) (0.150) (0.146) (0.156)

Income group

| Low-income | -8.285 | -2.894 | -4.792 | -2.875 | -6.429 | -5.190 | -4.689 | -10.834^**^ |
| --- | --- | --- | --- | --- | --- | --- | --- | --- |
|  | (5.230) | (4.892) | (4.766) | (4.823) | (5.113) | (4.970) | (4.791) | (5.264) |
| Lower-middle-income | 5.569 | 9.114^**^ | 6.814^*^ | 9.022^**^ | 6.152 | 7.515^*^ | 8.156^**^ | 2.900 |
|  | (4.112) | (3.941) | (3.879) | (3.871) | (4.116) | (3.980) | (3.826) | (4.191) |
| Upper-middle-income | 5.800 | 9.103^***^ | 7.374^**^ | 8.883^***^ | 6.508^*^ | 7.839^**^ | 7.956^**^ | 3.865 |
|  | (3.692) | (3.527) | (3.407) | (3.398) | (3.654) | (3.566) | (3.422) | (3.626) |
| *Observations* | 1898 | 1898 | 1881 | 1898 | 1773 | 1881 | 1915 | 1757 |
| *R*^2^(*overall*) | 0.30 | 0.29 | 0.29 | 0.29 | 0.27 | 0.26 | 0.31 | 0.28 |
| Hausman p-value | 0.00 | 0.00 | 0.00 | 0.00 | 0.00 | 0.00 | 0.00 | 0.00 |

Standard errors in parentheses

*Notes:* All state-capacity measures are standardized to mean 0 and SD 1.

^*^ p¡0.10, ^**^ p¡0.05, ^***^ p¡0.01

## Table A6: State capacity and Govt.health expenditure (RE model)

|  | (1) | (2) | (3) | (4) | (5) | (6) | (7) | (8) |
| --- | --- | --- | --- | --- | --- | --- | --- | --- |
| GHE per capita |  |  |  |  |  |  |  |  |
| Bureaucratic quality | 0.017 |  |  |  |  |  |  | -0.117^**^ |
|  | (0.056) |  |  |  |  |  |  | (0.060) |
| Control of corruption |  | 0.001 |  |  |  |  |  | -0.008 |
|  |  | (0.021) |  |  |  |  |  | (0.022) |
| Rule of law |  |  | 0.180^***^ |  |  |  |  | 0.069 |
|  |  |  | (0.045) |  |  |  |  | (0.046) |
| Civilian control of govt. |  |  |  | 0.062^**^ |  |  |  | -0.035 |
|  |  |  |  | (0.032) |  |  |  | (0.034) |
| Govt. effectiveness |  |  |  |  | 0.331^***^ |  |  | 0.280^***^ |
|  |  |  |  |  | (0.042) |  |  | (0.045) |
| Property rights |  |  |  |  |  | 0.154^***^ |  | 0.055 |
|  |  |  |  |  |  | (0.044) |  | (0.046) |
| State fragility Index |  |  |  |  |  |  | -0.383^***^ | -0.385^***^ |
|  |  |  |  |  |  |  | (0.041) | (0.043) |
| GDP growth (lag) | 0.007^***^ | 0.007^***^ | 0.008^***^ | 0.007^***^ | 0.007^***^ | 0.008^***^ | 0.006^***^ | 0.007^***^ |
|  | (0.002) | (0.002) | (0.002) | (0.002) | (0.002) | (0.002) | (0.002) | (0.002) |
| Population growth (lag) | 0.005 | 0.005 | 0.005 | 0.005 | 0.007 | 0.004 | 0.006 | 0.005 |
|  | (0.008) | (0.008) | (0.007) | (0.008) | (0.007) | (0.007) | (0.007) | (0.007) |
| Urban Population (lag) | 0.017^***^ | 0.017^***^ | 0.018^***^ | 0.017^***^ | 0.019^***^ | 0.016^***^ | 0.013^***^ | 0.014^***^ |
|  | (0.003) | (0.003) | (0.003) | (0.003) | (0.003) | (0.003) | (0.003) | (0.003) |
| Pop 65y and above | -0.017^*^ | -0.017^*^ | -0.017^*^ | -0.017^*^ | -0.023^**^ | -0.018^*^ | -0.017^*^ | -0.021^**^ |
|  | (0.010) | (0.010) | (0.010) | (0.010) | (0.010) | (0.010) | (0.009) | (0.009) |
| Polity2scores | 0.022^***^ | 0.022^***^ | 0.005 | 0.020^***^ | 0.017^***^ | 0.011^**^ | 0.015^***^ | 0.004 |
|  | (0.005) | (0.005) | (0.005) | (0.005) | (0.005) | (0.005) | (0.005) | (0.006) |
| Female edu attain (lag) | 0.529^***^ | 0.531^***^ | 0.524^***^ | 0.531^***^ | 0.499^***^ | 0.526^***^ | 0.491^***^ | 0.454^***^ |
|  | (0.015) | (0.015) | (0.014) | (0.015) | (0.015) | (0.015) | (0.015) | (0.015) |
| Income group |  |  |  |  |  |  |  |  |
| Low-income | -0.451^*^ | -0.484^**^ | -0.271 | -0.365 | -0.041 | -0.420^*^ | -0.138 | 0.045 |
|  | (0.250) | (0.231) | (0.237) | (0.239) | (0.234) | (0.237) | (0.231) | (0.254) |

Lower-middle-income -1.186^***^ -1.209^***^ -0.968^***^ -1.114^***^ -0.794^***^ -1.176^***^ -0.980^***^ -0.621^***^ (0.194) (0.186) (0.194) (0.191) (0.188) (0.189) (0.184) (0.202)

Upper-middle-income -1.065^***^ -1.085^***^ -0.909^***^ -1.034^***^ -0.708^***^ -1.051^***^ -0.911^***^ -0.672^***^ (0.170) (0.161) (0.166) (0.163) (0.162) (0.166) (0.160) (0.171)

| *Observations* | 1898 | 1898 | 1881 | 1898 | 1773 | 1881 | 1915 | 1757 |
| --- | --- | --- | --- | --- | --- | --- | --- | --- |
| *R*^2^(*overall*) | 0.83 | 0.82 | 0.83 | 0.83 | 0.84 | 0.82 | 0.84 | 0.86 |
| Hausman p-value | 0.00 | 0.00 | 0.00 | 0.00 | 0.00 | 0.00 | 0.00 | 0.00 |

Standard errors in parentheses

*Notes:* All state-capacity measures are standardized to mean 0 and SD 1.

^*^ p¡0.10, ^**^ p¡0.05, ^***^ p¡0.01

*Table A7: State capacity and Out-of-pocket health expenditure (FE model)*

|  | (1) | (2) | (3) | (4) | (5) | (6) | (7) | (8) | (9) |
| --- | --- | --- | --- | --- | --- | --- | --- | --- | --- |
| OOP %CHE |  |  |  |  |  |  |  |  |  |
| Bureaucratic quality | -2.586^**^ |  |  |  |  |  |  |  | -2.434^**^ |
|  | (1.103) |  |  |  |  |  |  |  | (1.226) |
| Control of corruption |  | 0.328 |  |  |  |  |  |  | 0.312 |
|  |  | (0.347) |  |  |  |  |  |  | (0.378) |
| Rule of law |  |  | -0.650 |  |  |  |  |  | 0.003 |
|  |  |  | (0.804) |  |  |  |  |  | (0.830) |
| Civilian control of govt. |  |  |  | 0.436 |  |  |  |  | 0.979 |
|  |  |  |  | (0.531) |  |  |  |  | (0.599) |
| Govt. effectiveness |  |  |  |  | -1.605^**^ |  |  |  | -1.094 |
| Property rights |  |  |  |  | (0.747) | -2.774^***^ |  |  | (0.784)  -2.214^**^ |
|  |  |  |  |  |  | (0.831) |  |  | (0.902) |
| State fragility Index |  |  |  |  |  |  | 1.012 |  | 0.801 |
|  |  |  |  |  |  |  | (0.697) |  | (0.763) |
| Aggregate SC measure |  |  |  |  |  |  |  | -0.730 |  |
|  |  |  |  |  |  |  |  | (0.536) |  |
| Polity2scores | -0.145^*^ | -0.147^*^ | -0.108 | -0.161^*^ | -0.170^*^ | -0.108 | -0.129 | -0.129 | -0.169^*^ |
|  | (0.085) | (0.085) | (0.098) | (0.087) | (0.090) | (0.089) | (0.085) | (0.093) | (0.102) |
| GDP growth (lag) | -0.045 | -0.045 | -0.046 | -0.046 | -0.037 | -0.036 | -0.042 | -0.035 | -0.029 |
|  | (0.032) | (0.032) | (0.033) | (0.032) | (0.033) | (0.033) | (0.032) | (0.033) | (0.033) |
| Population growth (lag) | -0.075 | -0.074 | -0.069 | -0.076 | -0.070 | -0.045 | -0.071 | -0.067 | -0.043 |
|  | (0.121) | (0.122) | (0.122) | (0.122) | (0.121) | (0.122) | (0.121) | (0.121) | (0.121) |

Urban Population (lag) -0.506^***^-0.522^***^-0.529^***^-0.518^***^-0.541^***^-0.510^***^-0.504^***^-0.535^***^-0.495^***^ (0.068) (0.068) (0.068) (0.068) (0.072) (0.068) (0.068) (0.072) (0.074)

Pop 65y and above 0.881^***^ 0.897^***^ 0.896^***^ 0.901^***^ 0.916^***^ 0.804^***^ 0.876^***^ 0.867^***^ 0.773^***^ (0.194) (0.194) (0.195) (0.194) (0.202) (0.196) (0.194) (0.204) (0.208)

Female edu attain (lag) -1.665^***^-1.651^***^-1.628^***^-1.646^***^-1.568^***^-1.524^***^-1.555^***^-1.515^***^-1.370^***^ (0.295) (0.295) (0.295) (0.295) (0.311) (0.296) (0.305) (0.317) (0.328)

Current health exp%GDP-0.629^***^-0.627^***^-0.594^***^-0.636^***^-0.666^***^-0.505^***^-0.598^***^-0.646^***^-0.608^***^

|  | (0.149) | (0.149) | (0.151) | (0.149) | (0.154) | (0.153) | (0.148) | (0.156) | (0.159) |
| --- | --- | --- | --- | --- | --- | --- | --- | --- | --- |
| *Observations* | 1898 | 1898 | 1881 | 1898 | 1773 | 1881 | 1915 | 1757 | 1757 |
| *R*^2^(*overall*) | 0.231 | 0.193 | 0.186 | 0.189 | 0.198 | 0.184 | 0.211 | 0.201 | 0.211 |

Standard errors in parentheses. SC measures are standardized to mean 0, SD 1. ^*^ *p <* 0*.*10, ^**^ *p <* 0*.*05, ^***^ *p <* 0*.*01

*Table A8: State capacity and Govt.health expenditure (FE model)*

|  | (1) | (2) | (3) | (4) | (5) | (6) | (7) | (8) | (9) |
| --- | --- | --- | --- | --- | --- | --- | --- | --- | --- |
| GHE per capita |  |  |  |  |  |  |  |  |  |
| Bureaucratic quality | -0.029 |  |  |  |  |  |  |  | -0.131^*^ |
|  | (0.067) |  |  |  |  |  |  |  | (0.070) |
| Control of corruption |  | -0.017 |  |  |  |  |  |  | -0.022 |
|  |  | (0.021) |  |  |  |  |  |  | (0.022) |
| Rule of law |  |  | 0.133^***^ |  |  |  |  |  | 0.054 |
|  |  |  | (0.047) |  |  |  |  |  | (0.048) |
| Civilian control of govt. |  |  |  | 0.044 |  |  |  |  | -0.027 |
| Govt. effectiveness |  |  |  | (0.032) | 0.312^***^ |  |  |  | (0.034)  0.273^***^ |
|  |  |  |  |  | (0.044) |  |  |  | (0.045) |
| Property rights |  |  |  |  |  | 0.137^***^ |  |  | 0.073 |
| State fragility Index |  |  |  |  |  | (0.048) | -0.323^***^ |  | (0.051)  -0.332^***^ |
| Aggregate SC measure |  |  |  |  |  |  | (0.041) | 0.175^***^ | (0.044) |
|  |  |  |  |  |  |  |  | (0.031) |  |
| Polity2scores | 0.018^***^ | 0.018^***^ | 0.002 | 0.016^***^ | 0.012^**^ | 0.008 | 0.013^**^ | 0.003 | 0.004 |

(0.005) (0.005) (0.006) (0.005) (0.005) (0.005) (0.005) (0.005) (0.006)

GDP growth (lag) 0.008^***^ 0.008^***^ 0.009^***^ 0.008^***^ 0.008^***^ 0.009^***^ 0.007^***^ 0.008^***^ 0.008^***^

| (0.002) | (0.002) | (0.002) | (0.002) | (0.002) | (0.002) | (0.002) | (0.002) | (0.002) |
| --- | --- | --- | --- | --- | --- | --- | --- | --- |
| Population growth (lag) -0.001 | -0.002 | -0.001 | -0.001 | 0.002 | -0.001 | -0.001 | -0.000 | -0.000 |
| (0.007) | (0.007) | (0.007) | (0.007) | (0.007) | (0.007) | (0.007) | (0.007) | (0.007) |
| Urban Population (lag) 0.002 | 0.001 | 0.004 | 0.002 | 0.006 | 0.003 | -0.003 | 0.005 | 0.002 |
| (0.004) | (0.004) | (0.004) | (0.004) | (0.004) | (0.004) | (0.004) | (0.004) | (0.004) |

Pop 65y and above -0.056^***^-0.056^***^-0.057^***^-0.056^***^-0.071^***^-0.052^***^-0.049^***^-0.061^***^-0.055^***^ (0.012) (0.012) (0.011) (0.012) (0.012) (0.011) (0.011) (0.012) (0.012)

Female edu attain (lag) 0.625^***^ 0.625^***^ 0.616^***^ 0.625^***^ 0.600^***^ 0.611^***^ 0.587^***^ 0.584^***^ 0.546^***^

|  | (0.017) | (0.017) | (0.017) | (0.017) | (0.018) | (0.017) | (0.018) | (0.018) | (0.018) |
| --- | --- | --- | --- | --- | --- | --- | --- | --- | --- |
| *Observations* | 1898 | 1898 | 1881 | 1898 | 1773 | 1881 | 1915 | 1757 | 1757 |
| *R*^2^(*overall*) | 0.662 | 0.663 | 0.686 | 0.676 | 0.718 | 0.673 | 0.704 | 0.746 | 0.761 |

Standard errors in parentheses. SC measures are standardized to mean 0, SD 1. ^*^ *p <* 0*.*10, ^**^ *p <* 0*.*05, ^***^ *p <* 0*.*01

*Table A9: State capacity and Out-of-pocket health expenditure, LMICs (FE model)*

|  | (1) | (2) | (3) | (4) | (5) | (6) | (7) | (8) | (9) |
| --- | --- | --- | --- | --- | --- | --- | --- | --- | --- |
| OOP %CHE |  |  |  |  |  |  |  |  |  |
| Bureaucratic quality | -5.348^**^ |  |  |  |  |  |  |  | -5.021^**^ |
| Control of corruption | (2.322) | 1.662^**^ |  |  |  |  |  |  | (2.505)  2.240^***^ |
|  |  | (0.666) |  |  |  |  |  |  | (0.707) |
| Rule of law |  |  | -1.437 |  |  |  |  |  | -0.601 |
| Civilian control of govt. |  |  | (1.183) | 0.711 |  |  |  |  | (1.227)  1.896^**^ |
| Govt. effectiveness |  |  |  | (0.844) | -2.666^*^ |  |  |  | (0.958)  -2.781^*^ |
|  |  |  |  |  | (1.458) |  |  |  | (1.533) |
| Property rights |  |  |  |  |  | -1.435 |  |  | 1.652 |
| State fragility Index |  |  |  |  |  | (1.320) | 4.868^***^ |  | (1.521)  6.446^***^ |
|  |  |  |  |  |  |  | (1.271) |  | (1.447) |
| Aggregate SC measure |  |  |  |  |  |  |  | -0.233 |  |
| Polity2scores | -0.286^**^ | -0.280^**^ | -0.198 | -0.283^**^ | -0.287^**^ | -0.264^**^ | -0.223^*^ | (0.947)  -0.264^**^ | -0.298^**^ |
|  | (0.120) | (0.119) | (0.138) | (0.122) | (0.127) | (0.124) | (0.118) | (0.134) | (0.146) |
| GDP growth (lag) | 0.026 | 0.019 | 0.010 | 0.016 | 0.045 | 0.017 | 0.031 | 0.035 | 0.026 |
|  | (0.066) | (0.065) | (0.066) | (0.066) | (0.067) | (0.066) | (0.064) | (0.068) | (0.067) |

Population growth (lag) -1.376^***^-1.447^***^-1.332^***^-1.455^***^-1.303^***^-1.353^***^-1.290^***^-1.360^***^-1.275^***^ (0.418) (0.417) (0.417) (0.419) (0.414) (0.416) (0.408) (0.424) (0.416)

Urban Population (lag) -0.686^***^-0.654^***^-0.694^***^-0.621^***^-0.717^***^-0.672^***^-0.597^***^-0.707^***^-0.722^***^ (0.167) (0.167) (0.168) (0.171) (0.181) (0.168) (0.162) (0.185) (0.185)

Pop 65y and above 2.932^***^ 3.573^***^ 3.960^***^ 3.755^***^ 3.423^***^ 3.731^***^ 3.932^***^ 3.275^***^ 2.443^**^

|  | (0.999) | (0.935) | (0.920) | (0.936) | (0.951) | (0.906) | (0.889) | (1.000) | (1.079) |
| --- | --- | --- | --- | --- | --- | --- | --- | --- | --- |
| Female edu attain (lag) | -0.199 | -0.664 | -0.617 | -0.880 | -0.496 | -0.583 | 0.093 | -0.428 | 1.593^*^ |
|  | (0.809) | (0.769) | (0.758) | (0.781) | (0.808) | (0.759) | (0.773) | (0.863) | (0.946) |
| Current health exp%GDP-1.207^***^-1.228^***^-1.135^***^-1.263^***^-1.168^***^-1.097^***^-1.020^***^-1.190^***^-1.127^***^ | | | | | | | | | |
|  | (0.243) | (0.243) | (0.246) | (0.247) | (0.250) | (0.255) | (0.242) | (0.263) | (0.266) |
| *Observations* | 658 | 658 | 658 | 658 | 621 | 658 | 675 | 605 | 605 |
| *R*^2^(*overall*) | 0.029 | 0.028 | 0.013 | 0.028 | 0.008 | 0.007 | 0.047 | 0.006 | 0.006 |

Standard errors in parentheses. SC measures are standardized to mean 0, SD 1. ^*^ *p <* 0*.*10, ^**^ *p <* 0*.*05, ^***^ *p <* 0*.*01

*Table A10: State capacity and Govt.health expenditure, LMICs (FE model)*

|  | (1) | (2) | (3) | (4) | (5) | (6) | (7) | (8) | (9) |
| --- | --- | --- | --- | --- | --- | --- | --- | --- | --- |
| GHE percapita |  |  |  |  |  |  |  |  |  |
| Bureaucratic quality | 0.042 |  |  |  |  |  |  |  | 0.037 |
| Control of corruption | (0.127) | 0.131^***^ |  |  |  |  |  |  | (0.128)  0.091^**^ |
|  |  | (0.036) |  |  |  |  |  |  | (0.036) |
| Rule of law |  |  | 0.105^*^ |  |  |  |  |  | 0.058 |
|  |  |  | (0.060) |  |  |  |  |  | (0.062) |
| Civilian control of govt. |  |  |  | 0.144^***^ |  |  |  |  | 0.071 |
| Govt. effectiveness |  |  |  | (0.045) | 0.392^***^ |  |  |  | (0.048)  0.287^***^ |
| Property rights |  |  |  |  | (0.074) | 0.228^***^ |  |  | (0.078)  0.188^**^ |
|  |  |  |  |  |  | (0.065) |  |  | (0.075) |
| State fragility Index |  |  |  |  |  |  | -0.086 |  | -0.081 |
| Aggregate SC measure |  |  |  |  |  |  | (0.069) | 0.282^***^ | (0.073) |
|  |  |  |  |  |  |  |  | (0.045) |  |
| Polity2scores | 0.023^***^ | 0.021^***^ | 0.007 | 0.018^***^ | 0.016^**^ | 0.011^*^ | 0.021^***^ | 0.003 | 0.008 |

(0.007) (0.006) (0.007) (0.007) (0.006) (0.006) (0.006) (0.007) (0.007)

GDP growth (lag) 0.012^***^ 0.012^***^ 0.016^***^ 0.011^***^ 0.013^***^ 0.015^***^ 0.012^***^ 0.012^***^ 0.012^***^

| (0.004) | (0.004) | (0.003) | (0.004) | (0.003) | (0.003) | (0.004) | (0.003) | (0.003) |
| --- | --- | --- | --- | --- | --- | --- | --- | --- |
| Population growth (lag) 0.001 | 0.002 | 0.006 | 0.002 | 0.008 | 0.006 | 0.007 | -0.007 | -0.004 |
| (0.023) | (0.023) | (0.021) | (0.023) | (0.021) | (0.021) | (0.023) | (0.021) | (0.021) |
| Urban Population (lag) -0.002 | -0.002 | 0.009 | 0.005 | 0.013 | 0.007 | -0.005 | 0.017^*^ | 0.016^*^ |
| (0.009) | (0.009) | (0.009) | (0.009) | (0.009) | (0.009) | (0.009) | (0.009) | (0.009) |

Pop 65y and above -0.146^***^-0.168^***^-0.183^***^-0.151^***^-0.182^***^-0.161^***^-0.185^***^-0.150^***^-0.157^***^ (0.055) (0.051) (0.047) (0.050) (0.048) (0.046) (0.049) (0.049) (0.055)

Female edu attain (lag) 0.658^***^ 0.672^***^ 0.619^***^ 0.636^***^ 0.603^***^ 0.607^***^ 0.665^***^ 0.531^***^ 0.544^***^

|  | (0.043) | (0.040) | (0.038) | (0.041) | (0.040) | (0.038) | (0.042) | (0.042) | (0.048) |
| --- | --- | --- | --- | --- | --- | --- | --- | --- | --- |
| *Observations* | 658 | 658 | 658 | 658 | 621 | 658 | 675 | 605 | 605 |
| *R*^2^(*overall*) | 0.466 | 0.464 | 0.536 | 0.506 | 0.574 | 0.548 | 0.450 | 0.625 | 0.622 |

Standard errors in parentheses. SC measures are standardized to mean 0, SD 1. ^*^ *p <* 0*.*10, ^**^ *p <* 0*.*05, ^***^ *p <* 0*.*01

## Table A11: State capacity (with 1 year lagged) and OOP expenditures (FE model)

|  | (1) | (2) | (3) | (4) | (5) | (6) | (7) |
| --- | --- | --- | --- | --- | --- | --- | --- |
| OOP %CHE |  |  |  |  |  |  |  |
| Bureaucratic quality (lag) | -2.376^∗∗^ |  |  |  |  |  |  |
|  | (1.002) |  |  |  |  |  |  |
| Control of corruption (lag) |  | 0.304 |  |  |  |  |  |
| Rule of law (lag) |  | (0.320) | -1.841^∗∗^ |  |  |  |  |
|  |  |  | (0.769) |  |  |  |  |
| Civilian control of govt. (lag) |  |  |  | -0.179 |  |  |  |
| Govt. effectiveness (lag) |  |  |  | (0.482) | -1.485^∗^ |  |  |
| Property rights (lag) |  |  |  |  | (0.786) | -3.178^∗∗∗^ |  |
|  |  |  |  |  |  | (0.769) |  |
| State fragility Index (lag) |  |  |  |  |  |  | 0.633 |
|  |  |  |  |  |  |  | (0.686) |
| Polity2scores | -0.147^∗^ | -0.147^∗^ | -0.057 | -0.139 | -0.184^∗∗^ | -0.113 | -0.135 |
|  | (0.085) | (0.085) | (0.095) | (0.087) | (0.094) | (0.088) | (0.085) |
| GDP growth (lag) | -0.044 | -0.045 | -0.042 | -0.045 | -0.072^∗∗^ | -0.027 | -0.043 |
|  | (0.032) | (0.032) | (0.033) | (0.032) | (0.034) | (0.033) | (0.032) |
| Population growth (lag) | -0.073 | -0.073 | -0.065 | -0.078 | 0.046 | -0.038 | -0.070 |
|  | (0.121) | (0.122) | (0.122) | (0.122) | (0.122) | (0.122) | (0.121) |

Urban Population (lag) -0.506^∗∗∗^ -0.522^∗∗∗^ -0.539^∗∗∗^ -0.523^∗∗∗^ -0.577^∗∗∗^ -0.506^∗∗∗^ -0.510^∗∗∗^

(0.068) (0.068) (0.068) (0.068) (0.078) (0.068) (0.068)

Pop 65y and above 0.888^∗∗∗^ 0.897^∗∗∗^ 0.910^∗∗∗^ 0.898^∗∗∗^ 0.837^∗∗∗^ 0.796^∗∗∗^ 0.892^∗∗∗^

(0.194) (0.194) (0.194) (0.194) (0.215) (0.195) (0.194)

Female edu attain (lag) -1.674^∗∗∗^ -1.635^∗∗∗^ -1.593^∗∗∗^ -1.646^∗∗∗^ -1.505^∗∗∗^ -1.480^∗∗∗^ -1.599^∗∗∗^

(0.295) (0.295) (0.295) (0.295) (0.336) (0.296) (0.307)

Current health exp%GDP -0.627^∗∗∗^ -0.624^∗∗∗^ -0.594^∗∗∗^ -0.624^∗∗∗^ -0.677^∗∗∗^ -0.495^∗∗∗^ -0.602^∗∗∗^

|  | (0.149) | (0.149) | (0.151) | (0.149) | (0.161) | (0.152) | (0.149) |
| --- | --- | --- | --- | --- | --- | --- | --- |
| *Observations* | 1898 | 1898 | 1881 | 1898 | 1664 | 1881 | 1915 |
| *R*^2^(*overall*) | 0.229 | 0.193 | 0.208 | 0.201 | 0.198 | 0.185 | 0.205 |

Standard errors in parentheses

^∗^ *p <* 0*.*10, ^∗∗^ *p <* 0*.*05, ^∗∗∗^ *p <* 0*.*01

| *Table A12: State capacity (with 1 year lagged) and GHE (FE model)* | | | | | | | |
| --- | --- | --- | --- | --- | --- | --- | --- |
|  | (1) | (2) | (3) | (4) | (5) | (6) | (7) |
| GHE per capita |  |  |  |  |  |  |  |
| Bureaucratic quality (lag) | 0.011 |  |  |  |  |  |  |
| Control of corruption (lag) | (0.061) | -0.043^∗∗^ |  |  |  |  |  |
| Rule of law (lag) |  | (0.019) | 0.102^∗∗^ |  |  |  |  |
| Civilian control of govt. (lag) |  |  | (0.046) | 0.070^∗∗^ |  |  |  |
| Govt. effectiveness (lag) |  |  |  | (0.029) | 0.264^∗∗∗^ |  |  |
| Property rights (lag) |  |  |  |  | (0.045) | 0.162^∗∗∗^ |  |
| State fragility Index (lag) |  |  |  |  |  | (0.045) | -0.288^∗∗∗^ |
| Polity2scores | 0.018^∗∗∗^ | 0.018^∗∗∗^ | 0.005 | 0.015^∗∗∗^ | 0.009 | 0.008 | (0.041)  0.013^∗∗∗^ |
|  | (0.005) | (0.005) | (0.006) | (0.005) | (0.005) | (0.005) | (0.005) |
| GDP growth (lag) | 0.008^∗∗∗^ | 0.008^∗∗∗^ | 0.009^∗∗∗^ | 0.008^∗∗∗^ | 0.007^∗∗∗^ | 0.008^∗∗∗^ | 0.007^∗∗∗^ |
|  | (0.002) | (0.002) | (0.002) | (0.002) | (0.002) | (0.002) | (0.002) |
| Population growth (lag) | -0.002 | -0.002 | -0.001 | -0.001 | -0.003 | -0.002 | -0.002 |
|  | (0.007) | (0.007) | (0.007) | (0.007) | (0.007) | (0.007) | (0.007) |
| Urban Population (lag) | 0.001 | 0.001 | 0.004 | 0.002 | 0.005 | 0.003 | -0.002 |
|  | (0.004) | (0.004) | (0.004) | (0.004) | (0.004) | (0.004) | (0.004) |
| Pop 65y and above -0.056^∗∗∗^ -0.056^∗∗∗^ -0.057^∗∗∗^ -0.056^∗∗∗^ -0.083^∗∗∗^ -0.052^∗∗∗^ -0.050^∗∗∗^ | | | | | | | |
|  | (0.012) | (0.012) | (0.011) | (0.012) | (0.012) | (0.011) | (0.012) |
| Female edu attain (lag) | 0.625^∗∗∗^ | 0.623^∗∗∗^ | 0.616^∗∗∗^ | 0.624^∗∗∗^ | 0.599^∗∗∗^ | 0.608^∗∗∗^ | 0.589^∗∗∗^ |
|  | (0.017) | (0.017) | (0.017) | (0.017) | (0.019) | (0.017) | (0.018) |
| *Observations* | 1898 | 1898 | 1881 | 1898 | 1664 | 1881 | 1915 |
| *R*^2^(*overall*) | 0.669 | 0.657 | 0.680 | 0.680 | 0.687 | 0.675 | 0.701 |

Standard errors in parentheses

^∗^ *p <* 0*.*10, ^∗∗^ *p <* 0*.*05, ^∗∗∗^ *p <* 0*.*01

*Table A13: Tercile Analyses of State capacity and health expenditure (FE model)*

|  | (1) OOP | (2) GHE |
| --- | --- | --- |
| Aggregate SC measure tercile Terciles =2 | -1.532^∗∗^ | 0.136^∗∗∗^ |
|  | (0.675) | (0.040) |
| Terciles =3 | -1.194 | -0.044 |
| Polity2scores | (1.509)  -0.152^∗^ | (0.089)  0.009^∗^ |
|  | (0.090) | (0.005) |
| GDP growth (lag) | -0.038 | 0.009^∗∗∗^ |
|  | (0.033) | (0.002) |
| Population growth (lag) | -0.071 | -0.002 |
|  | (0.123) | (0.007) |
| Urban Population (lag) | -0.536^∗∗∗^ | 0.005 |
| Pop 65y and above | (0.072)  0.844^∗∗∗^ | (0.004)  -0.061^∗∗∗^ |
| Female edu attain (lag) | (0.205)  -1.496^∗∗∗^ | (0.012)  0.598^∗∗∗^ |
| Current health exp%GDP | (0.317)  -0.665^∗∗∗^ | (0.018) |
|  | (0.155) |  |
| *Observations* | 1757 | 1757 |
| *R*^2^(*overall*) | 0.181 | 0.652 |
| Standard errors in parentheses |  |  |
| ^∗^ *p <* 0*.*10, ^∗∗^ *p <* 0*.*05, ^∗∗∗^ *p <* 0*.*01 |  |  |

# Correlation matrix:

Appendix Tables ([A14](#_bookmark8)) to ([A16](#_bookmark9)) present the correlation matrix for full sample and sub- samples (high-income countries (HICs) and LMICs). The results show that most state capacity indicators —such as bureaucratic quality, control of corruption, rule of law, government effectiveness, and property rights— are strongly and positively correlated with each other, confirming that these dimensions capture overlapping institutional quality aspects.

The negative correlation between GDP growth and state capacity indicators in the full sample primarily reflects structural differences between HICs and LMICs. As shown in appendix Tables [A14](#_bookmark8) to [A16](#_bookmark9), this relationship is heterogeneous across income groups. In HICs —where governance quality is already high and long-run growth rates tend to be low— GDP growth is negatively correlated with state-capacity indicators. In contrast, in LMICs, the correlations become positive, suggesting that improvements in state capacity are associated with faster economic growth in these settings. These patterns reflect the fact that high-capacity, mature economies grow slowly, whereas governance improvements in developing countries often coincide with or facilitate economic expansion.

Overall, the correlation structure for HICs indicates a tight clustering among governance indicators, consistent with advanced institutional development, but also highlights the limited role of short-term growth fluctuations in shaping governance performance in these settings.

43

*Table A14: Correlation matrix (Full sample)*

|  | | (1) | (2) | (3) | (4) | (5) | (6) | (7) | (8) | (9) | (10) | (11) | (12) | (13) | (14) | (15) |
| --- | --- | --- | --- | --- | --- | --- | --- | --- | --- | --- | --- | --- | --- | --- | --- | --- |
| Bureaucratic quality (1) |  | 1.00 |  |  |  |  |  |  |  |  |  |  |  |  |  |  |
| Corruption (2) |  | 0.72 | 1.00 |  |  |  |  |  |  |  |  |  |  |  |  |  |
| Rule of law (3) |  | 0.73 | 0.68 | 1.00 |  |  |  |  |  |  |  |  |  |  |  |  |
| Civilian control of govt. | (4) | 0.65 | 0.57 | 0.73 | 1.00 |  |  |  |  |  |  |  |  |  |  |  |
| Govt. effectiveness (5) | | 0.89 | 0.82 | 0.81 | 0.73 | 1.00 |  |  |  |  |  |  |  |  |  |  |
| Property rights (6) | | 0.48 | 0.47 | 0.58 | 0.53 | 0.53 | 1.00 |  |  |  |  |  |  |  |  |  |
| State fragility Index (7) | | -0.70 | -0.60 | -0.74 | -0.79 | -0.80 | -0.64 | 1.00 |  |  |  |  |  |  |  |  |
| Polity2scores (8) | | 0.41 | 0.36 | 0.60 | 0.38 | 0.38 | 0.64 | -0.41 | 1.00 |  |  |  |  |  |  |  |
| GDP growth (9) | | -0.20 | -0.20 | -0.21 | -0.16 | -0.20 | -0.22 | 0.24 | -0.24 | 1.00 |  |  |  |  |  |  |
| GDP per capita (10) | | 0.67 | 0.63 | 0.55 | 0.57 | 0.77 | 0.25 | -0.67 | 0.03 | -0.12 | 1.00 |  |  |  |  |  |
| Population growth (11) | | -0.25 | -0.18 | -0.28 | -0.29 | -0.23 | -0.48 | 0.37 | -0.48 | 0.23 | 0.07 | 1.00 |  |  |  |  |
| Urban Population (12) | | 0.51 | 0.50 | 0.44 | 0.47 | 0.61 | 0.36 | -0.70 | 0.08 | -0.16 | 0.68 | -0.09 | 1.00 |  |  |  |
| Pop 65y and above (13) | | 0.60 | 0.56 | 0.64 | 0.59 | 0.67 | 0.66 | -0.74 | 0.53 | -0.29 | 0.46 | -0.61 | 0.46 | 1.00 |  |  |
| Female edu attain (14) | | 0.68 | 0.50 | 0.56 | 0.65 | 0.71 | 0.51 | -0.80 | 0.32 | -0.17 | 0.60 | -0.37 | 0.69 | 0.67 | 1.00 |  |
| Current health exp%GDP (15) | | 0.39 | 0.50 | 0.53 | 0.44 | 0.47 | 0.50 | -0.47 | 0.55 | -0.30 | 0.25 | -0.37 | 0.33 | 0.61 | 0.40 | 1.00 |

44

*Table A15: Correlation matrix (HICs sample)*

|  |  | (1) | (2) | (3) | (4) | (5) | (6) | (7) | (8) | (9) | (10) | (11) | (12) | (13) | (14) | (15) |
| --- | --- | --- | --- | --- | --- | --- | --- | --- | --- | --- | --- | --- | --- | --- | --- | --- |
| Bureaucratic quality (1) |  | 1.00 |  |  |  |  |  |  |  |  |  |  |  |  |  |  |
| Corruption (2) |  | 0.79 | 1.00 |  |  |  |  |  |  |  |  |  |  |  |  |  |
| Rule of law (3) |  | 0.76 | 0.70 | 1.00 |  |  |  |  |  |  |  |  |  |  |  |  |
| Civilian control of govt. | (4) | 0.59 | 0.55 | 0.65 | 1.00 |  |  |  |  |  |  |  |  |  |  |  |
| Govt. effectiveness (5) | | 0.89 | 0.85 | 0.79 | 0.64 | 1.00 |  |  |  |  |  |  |  |  |  |  |
| Property rights (6) | | 0.43 | 0.43 | 0.59 | 0.41 | 0.41 | 1.00 |  |  |  |  |  |  |  |  |  |
| State fragility Index (7) | | -0.62 | -0.60 | -0.75 | -0.74 | -0.72 | -0.54 | 1.00 |  |  |  |  |  |  |  |  |
| Polity2scores (8) | | 0.43 | 0.37 | 0.65 | 0.38 | 0.38 | 0.79 | -0.46 | 1.00 |  |  |  |  |  |  |  |
| GDP growth (9) | | -0.22 | -0.19 | -0.25 | -0.19 | -0.19 | -0.25 | 0.24 | -0.29 | 1.00 |  |  |  |  |  |  |
| GDP per capita (10) | | 0.59 | 0.59 | 0.42 | 0.43 | 0.68 | 0.02 | -0.52 | -0.09 | -0.05 | 1.00 |  |  |  |  |  |
| Population growth (11) | | -0.07 | -0.07 | -0.19 | -0.19 | -0.04 | -0.51 | 0.21 | -0.52 | 0.22 | 0.34 | 1.00 |  |  |  |  |
| Urban Population (12) | | 0.35 | 0.46 | 0.31 | 0.16 | 0.44 | 0.04 | -0.43 | -0.07 | -0.08 | 0.57 | 0.26 | 1.00 |  |  |  |
| Pop 65y and above (13) | | 0.48 | 0.48 | 0.56 | 0.48 | 0.52 | 0.71 | -0.68 | 0.60 | -0.29 | 0.21 | -0.56 | 0.15 | 1.00 |  |  |
| Female edu attain (14) | | 0.58 | 0.50 | 0.48 | 0.49 | 0.59 | 0.47 | -0.59 | 0.32 | -0.18 | 0.40 | -0.19 | 0.39 | 0.56 | 1.00 |  |
| Current health exp%GDP (15) | | 0.47 | 0.54 | 0.60 | 0.45 | 0.49 | 0.67 | -0.51 | 0.65 | -0.35 | 0.10 | -0.38 | 0.12 | 0.66 | 0.41 | 1.00 |
| *HICs*: High Income Countries | |  |  |  |  |  |  |  |  |  |  |  |  |  |  |  |

45

*Table A16: Correlation matrix (LMICs sample)*

|  |  | (1) | (2) | (3) | (4) | (5) | (6) | (7) | (8) | (9) | (10) | (11) | (12) | (13) | (14) | (15) |
| --- | --- | --- | --- | --- | --- | --- | --- | --- | --- | --- | --- | --- | --- | --- | --- | --- |
| Bureaucratic quality (1) |  | 1.00 |  |  |  |  |  |  |  |  |  |  |  |  |  |  |
| Corruption (2) |  | 0.10 | 1.00 |  |  |  |  |  |  |  |  |  |  |  |  |  |
| Rule of law (3) |  | 0.21 | 0.19 | 1.00 |  |  |  |  |  |  |  |  |  |  |  |  |
| Civilian control of govt. | (4) | 0.32 | 0.23 | 0.55 | 1.00 |  |  |  |  |  |  |  |  |  |  |  |
| Govt. effectiveness (5) | | 0.69 | 0.32 | 0.47 | 0.48 | 1.00 |  |  |  |  |  |  |  |  |  |  |
| Property rights (6) | | 0.19 | 0.31 | 0.24 | 0.36 | 0.43 | 1.00 |  |  |  |  |  |  |  |  |  |
| State fragility Index (7) | | -0.44 | -0.29 | -0.31 | -0.46 | -0.61 | -0.58 | 1.00 |  |  |  |  |  |  |  |  |
| Polity2scores (8) | | 0.24 | 0.10 | 0.45 | 0.33 | 0.24 | 0.28 | -0.33 | 1.00 |  |  |  |  |  |  |  |
| GDP growth (9) | | 0.06 | -0.03 | 0.16 | 0.12 | 0.05 | -0.01 | 0.08 | 0.02 | 1.00 |  |  |  |  |  |  |
| GDP per capita (10) | | 0.44 | 0.02 | -0.06 | 0.10 | 0.48 | 0.23 | -0.53 | 0.08 | -0.02 | 1.00 |  |  |  |  |  |
| Population growth (11) | | -0.37 | -0.16 | 0.06 | -0.03 | -0.35 | -0.19 | 0.45 | -0.11 | 0.13 | -0.38 | 1.00 |  |  |  |  |
| Urban Population (12) | | 0.13 | 0.07 | -0.21 | 0.08 | 0.20 | 0.33 | -0.48 | 0.08 | -0.10 | 0.71 | -0.31 | 1.00 |  |  |  |
| Pop 65y and above (13) | | 0.44 | 0.23 | 0.03 | 0.05 | 0.54 | 0.26 | -0.53 | 0.13 | -0.08 | 0.70 | -0.64 | 0.42 | 1.00 |  |  |
| Female edu attain (14) | | 0.50 | 0.05 | 0.05 | 0.25 | 0.43 | 0.21 | -0.57 | 0.28 | 0.05 | 0.72 | -0.47 | 0.49 | 0.57 | 1.00 |  |
| Current health exp%GDP (15) | | -0.27 | 0.03 | -0.03 | 0.05 | -0.17 | -0.03 | -0.06 | 0.18 | -0.05 | 0.01 | 0.01 | 0.25 | 0.08 | 0.00 | 1.00 |

*Table A17: Overview of Governance and Institutional Quality Datasets*

| **Dataset source** | | **Respondent type** | **Instrument design** | **Aggregation**  **/ Scaling** | **Update frequency** | | **Known biases / limitations** |
| --- | --- | --- | --- | --- | --- | --- | --- |
| *Quality* | *of* | Experts | Three-part | Regression- | Standard, | | Measurement |
| *Governance* | | selected for | questionnaire: | adjusted | Basic, | and | error due to |
| *Dataset* | | contextual | (1) | expert | OECD | | subjective expert |
| *(QoG)* | | subject- | bureaucratic | ratings using | datasets | | perceptions and |
|  | | matter | structure and | anchoring | updated | | scale differences; |
|  | | knowledge | behaviour; | vignettes and | annually | | mitigated in |
|  | |  | (2) anchoring | Item Response | (January); | | Wave 3 via |
|  | |  | vignettes | Theory (IRT) | European | | anchoring |
|  | |  | for scale | to correct for | QoG | Index | vignettes and |
|  | |  | adjustment; | systematic | every 3 years | | IRT |
|  | |  | (3) expert | reliability |  | |  |
|  | |  | characteristics | differences |  | |  |
| *Varieties of* | | Country | Five indicator | Indicators | Updated | | Expert judgment |
| *Democracy* | | experts | types: (A) | aggregated | annually | | subjectivity and |
| *(V-Dem)* | | with subject | factual (V- | via Bayesian | (March) | | cross-country |
|  | | expertise, | Dem team); | factor analysis |  | | bias; mitigated |
|  | | impartiality, | (B) factual | accounting for |  | | through |
|  | | and diversity | (country | measurement |  | | redundancy, |
|  | |  | coordinators); | error; latent |  | | calibration, and |
|  | |  | (C) evaluative | scores |  | | aggregation |
|  | |  | (expert | combined |  | | methods |
|  | |  | ratings); | across |  | |  |
|  | |  | (D) composite | components |  | |  |
|  | |  | indices; (E) |  |  | |  |
|  | |  | secondary |  |  | |  |
|  | |  | data |  |  | |  |
| *International* | | PRS staff | Political | Composite | Updated | | Subjectivity |
| *Country* | | coders | Risk Ratings | Risk Rating = | monthly | | and limited |
| *Risk Guide* | |  | constructed | Political (100) |  | | transparency in |
| *(ICRG–* | |  | by assigning | + Financial |  | | internal coding |
| *PRS)* | |  | points (0– | (50) + |  | | procedures |
|  | |  | 100) to 12 | Economic (50) |  | |  |
|  | |  | components | risk indices, |  | |  |
|  | |  | (e.g., | normalized for |  | |  |
|  | |  | government | comparability |  | |  |
|  | |  | stability, |  |  | |  |
|  | |  | corruption, |  |  | |  |
|  | |  | military in |  |  | |  |
|  | |  | politics, law |  |  | |  |
|  | |  | and order) |  |  | |  |
| *Polity* | *V* | No direct | Regime | Polity score | Updated | | Historical |
| *Project* | | respondents; | characteristics | = Democracy | annually | | inconsistencies; |
|  | | coded by | coded annually | (DEMOC) |  | | potential under |
|  | | researchers | for executive | – Autocracy |  | | representation of |
|  | |  | recruitment, | (AUTOC); |  | | informal power |
|  | |  | constraints, | derived from |  | | structures |
|  | |  | participation | component |  | |  |
|  | |  | regulation, | indices |  | |  |
|  | |  | and |  |  | |  |
|  | |  | competitiveness |  |  | |  |

*Note:* Table summarises key characteristics of major governance and institutional quality datasets used in the study, including data sources, instrument design, aggregation techniques, update cycles, and known limitations. All datasets are publicly accessible and widely used in cross-country empirical analyses of governance and state capacity. 46

## Table A18: Sample of Countries

| **H/UMICs*** |  |  |  |
| --- | --- | --- | --- |
| 1 Albania | 37 Iceland | 73 Slovakia | 108 Guinea-Bissau |
| 2 Argentina | 38 Iraq | 74 Slovenia | 109 Haiti |
| 3 Armenia | 39 Ireland | 75 Somalia | 110 Honduras |
| 4 Australia | 40 Israel | 76 South Africa | 111 India |
| 5 Austria | 41 Italy | 77 South Korea | 112 Indonesia |
| 6 Azerbaijan | 42 Jamaica | 78 Spain | 113 Iran |
| 7 Bahamas | 43 Japan | 79 Suriname | 114 Kenya |
| 8 Bahrain | 44 Jordan | 80 Sweden | 115 Lebanon |
| 9 Belarus | 45 Kazakhstan | 81 Switzerland | 116 Liberia |
| 10 Belgium | 46 Korea, DPR | 82 Syria | 117 Madagascar |
| 11 Botswana | 47 Kuwait | 83 Taiwan | 118 Malawi |
| 12 Brazil | 48 Latvia | 84 Thailand | 119 Mali |
| 13 Brunei | 49 Libya | 85 Trinidad-&-  Tobago | 120 Mongolia |
| 14 Bulgaria | 50 Lithuania | 86 Turkey | 121 Morocco |
| 15 Canada | 51 Luxembourg | 87 UAE | 122 Mozambique |
| 16 Chile | 52 Malaysia | 88 Uganda | 123 Myanmar |
| 17 China | 53 Malta | 89 Ukraine | 124 Nicaragua |
| 18 Colombia | 54 Mexico | 90 United Kingdom | 125 Niger |
| 19 Costa Rica | 55 Moldova | 91 United States | 126 Nigeria |
| 20 Croatia | 56 Namibia | 92 Uruguay | 127 Pakistan |
| 21 Cuba | 57 Netherlands | **LMICs**** | 128 Papua-New-  Guinea |
| 22 Cyprus | 58 New Zealand | 93 Algeria | 129 Philippines |
| 23 Czech Republic | 59 Norway | 94 Angola | 130 Senegal |
| 24 Denmark | 60 Oman | 95 Bangladesh | 131 Sierra Leone |
| 25 Dominican  Republic | 61 Panama | 96 Bolivia | 132 Sri Lanka |
| 26 Ecuador | 62 Paraguay | 97 Burkina Faso | 133 Sudan |
| 27 Estonia | 63 Peru | 98 Cameroon | 134 Tanzania |
| 28 Finland | 64 Poland | 99 Congo | 135 Togo |
| 29 France | 65 Portugal | 100 Congo, DR | 136 Tunisia |
| 30 Gabon | 66 Qatar | 101 Cote d’Ivoire | 137 Venezuela |
| 31 Germany | 67 Romania | 102 Egypt | 138 Vietnam |
| 32 Greece | 68 Russia | 103 El Salvador | 139 Yemen |
| 33 Guatemala | 69 Saudi Arabia | 104 Ethiopia | 140 Zambia |
| 34 Guyana | 70 Serbia | 105 Gambia | 141 Zimbabwe |
| 35 Hong Kong | 71 Serbia-&-  Montenegro | 106 Ghana |  |
| 36 Hungary | 72 Singapore | 107 Guinea |  |

^∗^ High-income and upper-middle-income, ^∗∗^ Low-income and Lower-middle-income countries

47
