# Supplementary material for: State capacity and health system financing: a cross-country analysis
Source: BMJ Glob Health. 2026 Mar 24;11(3):e020101. doi: 10.1136/bmjgh-2025-020101 (PMC13034253; doi:10.1136/bmjgh-2025-020101)
Supplement: online supplemental file 2 [file bmjgh-11-3-s002.docx]

### BMJ Global Health Author Reflexivity Statement

Adapted from Morton, B., Vercueil, A., Masekela, R., Heinz, E., Reimer, L., Saleh, S., Kalinga, C., Seekles, M., Biccard, B., Chakaya, J., Abimbola, S., Obasi, A. and Oriyo, N. (2022), Consensus statement on measures to promote equitable authorship in the publication of research from international partnerships. Anaesthesia, 77: 264-276. <https://doi.org/10.1111/anae.15597>

| **Study conceptualisation** | |
| --- | --- |
| 1. How does this study address local research and policy priorities? | The scope of the study is not around local research and policy priorities but on addressing cross-country/global questions around state capacity and health financing outcomes. However, it establishes methodological and conceptual approaches for explaining state capacity which can be adapted for answering local questions |
| 1. How were local researchers involved in study design? | Two LMIC researchers were involved as co-authors. The senior researcher (the lead politics specialist in the author team) contributed to the study’s conceptualisation and identification of variables and in finalising the study design. The ECR co-author contributed to data analysis and literature review. |
| **Research management** | |
| 1. How has funding been used to support the local research team(s)? | Funding (as a part of the NIHR Grant supporting the wider research project) has been used to support salaries of the ECR, and time buy0out of the senior researcher for activities under the project associated with production of outputs such as the present article manuscript. |
| **Data acquisition and analysis** | |
| 1. How are research staff who conducted data collection acknowledged? | N/A |
| 1. How have members of the research partnership been provided with access to study data? | All data used in the article were public access and/or proprietary (acquired through project funds) and was hosted in a secure data sharing platform governed by the projects’ data management systems. All co-authors had access to the data, the software programmes and results. |
| 1. How were data used to develop analytical skills within the partnership? | Senior co-authors had provided useful mentoring support to the ECRs in econometric approaches for data analysis through sharing of codes, allowing to develop test codes and programs and run analytical models |
| **Data interpretation** | |
| 1. How have research partners collaborated in interpreting study data? | Bi-weekly meetings were organised by the co-author team where the results were discussed, revised and finalised with all members, including LMIC researchers, assuming key roles |
| **Drafting and revising for intellectual content** | |
| 1. How were research partners supported to develop writing skills? | The senior author needed no support, being an established researcher himself, The ECR was provided feedback through comments on drafts produced |
| 1. How will research products be shared to address local needs? | Through blogs and newspaper op-eds |
| **Authorship** | |
| 1. How is the leadership, contribution and ownership of this work by LMIC researchers recognised within the authorship? | All credits have been duly acknowledged and declared, with leadership roles of the senior co-author (ZM) clearly stated in conceptualising, contributing to interpretation of results, drafting and revising the manuscript; the ECR (KB) role is also clarified through key roles I literature review and data analysis support, apart from reviewing draft. |
| 1. How have early career researchers across the partnership been included within the authorship team? | See (10) |
| 1. How has gender balance been addressed within the authorship? | We have one female co-author in the author team |
| **Training** | |
| 1. How has the project contributed to training of LMIC researchers? | See (6) above. The ECR co-author was constantly supported for developing her key analytical and theoretical skills through a sustained process of feedback, independent roles and exposure to seminars where drafts were presented |
| **Infrastructure** | |
| 1. How has the project contributed to improvements in local infrastructure? | N/A |
| **Governance** | |
| 1. What safeguarding procedures were used to protect local study participants and researchers? | N/A as entirely based on multicountry, secondary aggregate databases |
